# Publisher Correction: Learning interpretable cellular and gene signature embeddings from single-cell transcriptomic data

**DOI:** 10.1038/s41467-021-26140-y

**Published:** 2021-10-01

**Authors:** Yifan Zhao, Huiyu Cai, Zuobai Zhang, Jian Tang, Yue Li

**Affiliations:** 1 School of Computer Science, McGill University, Montreal, QC Canada; 2 Department of Machine Intelligence, Peking University, Beijing, China; 3 School of Computer Science, Fudan University, Shanghai, China; 4 HEC Montreal, Montreal, QC Canada; 5 Present Address: Harvard-MIT Health Sciences and Technology, Cambridge, MA USA

**Keywords:** Functional clustering, Machine learning

Correction to: *Nature Communications* 10.1038/s41467-021-25534-2, published online 6 September 2021.

In the original PDF version of this Article, there was an error in the code within the 'Methods' subsection ‘scETM software’. The original text read:

“from scETM import scETM,

UnsupervisedTrainermodel = scETM(adata.n_

vars, adata.obs.batch_indices.nunique())

trainer = UnsupervisedTrainer(model, adata)

trainer.train(save_model_ckpt = False)model.

get_all_embeddings_and_nll(adata)”

The correct format is:

“from scETM import scETM, UnsupervisedTrainer

model = scETM(adata.n_vars, adata.obs.batch_indices.nunique())

trainer = UnsupervisedTrainer(model, adata)

trainer.train(save_model_ckpt = False)

model.get_all_embeddings_and_nll(adata)”

This has been corrected in the PDF version of the Article; the HTML version was correct at the time of publication.

