# Supplementary material for: Learning interpretable cellular and gene signature embeddings from single-cell transcriptomic data
Source: Nat Commun. 2021 Sep 6;12:5261. doi: 10.1038/s41467-021-25534-2 (PMC8421403; doi:10.1038/s41467-021-25534-2)
Supplement: Supplementary file 1 — Supplementary Information [file 41467_2021_25534_MOESM1_ESM.pdf]

Supplementary Information: Learning interpretable cellular and gene signature embeddings from single-cell transcriptomic data

Yifan Zhao[1,2,†], **Huiyu Cai**[3,†], **Zuobai Zhang**[4], **Jian Tang**[5,*], **Yue Li**[1,*]

[1]School of Computer Science, McGill University, [2]Present address: Harvard-MIT Health Sciences and Technology, [3]Department of Machine Intelligence, Peking University, [4]School of Computer Science, Fudan University [5]HEC Montreal

[†]Equal contribution [*]Correspondence: `jian.tang@hec.ca`, `yueli@cs.mcgill.ca`

1 Supplementary Methods

1.1 Data processing

All of the single-cell datasets used in this study are from publicly available repositories or data portals. We describe below the acquisition and quality control (QC) for each of the datasets used in the current work.

1.1.1 Human pancreatic islet

We obtained the human pancreatic islet dataset and the ground truth cell type labels from Satija Lab at the following link: `https://satijalab.org/seurat/v3.0/integration.html` (accessed 1 Dec 2020), originally deposited by Stuart *et al.* [1]. This dataset is a compilation of scRNA-seq data from five studies which can be accessed using the following Gene Expression Omnibus (GEO) accession numbers: GSE81076 (CelSeq), GSE85241 (CelSeq2), GSE86469 (Fluidigm C1), E-MTAB-5061 (SMART-Seq2), and GSE84133 (inDrop). A QC step was conducted by [1], and no additional QC was performed. In our benchmarking experiment, we use the different scRNA-seq technologies as the batch variable.

1.1.2 Mouse pancreatic islet

We obtained the mouse pancreatic islet data and ground truth cell type labels from GSE84133 (inDrops) without conducting additional QC. There are 1,886 mouse cells from two mice of different strains, ICR and C57BL/6 [2]. The cell counts from the two strains are of approximately equal proportions. In our benchmarking experiment, we treated the mouse strain as the batch variable because of the different genetic backgrounds.

1.1.3 Major Depressive Disorder (MDD)

We obtained the 10X Genomics-based MDD snRNA-seq dataset with ground truth cell type labels from GSE144136. A strict QC step was conducted in the original empirical study by [3], where cells with fewer than 110 detected genes were removed. The top 0.5% of cells based on the total number of UMI (unique molecular identifiers) detected in each cell were also excluded because they are likely to be multiplets rather than single nuclei. No additional QC was performed. The MDD dataset consists of 78,886 cells from the dorsolateral prefrontal cortex of 34 male participants. The participants in the control group (n=17) who died due to natural cause and case group (n=17) who died by suicide were matched for age (18-87 years), post-mortem interval (12-93h) and brain pH (6-7.01) [3]. The number of cells from each donor is approximately the same.

1.1.4 Alzheimer's Disease (AD)

We obtained the droplet-based AD snRNA-seq data and the corresponding ground truth cell type labels from Synapse (`https://www.synapse.org/#!Synapse:syn18485175`) under the doi `10.7303/syn18485175`, and the metadata from `https://www.synapse.org/#!Synapse:syn3157322`. A strict QC step based on UMI counts and mitochondrial ratio values was conducted in the original empirical study by Mathys *et al.* [4]. The AD dataset consists of 70,634 cells from the prefrontal cortex of 48 individuals, both male and female, in the Religious Order Study (ROS) or the Rush Memory and Aging Project (MAP), two longitudinal cohort studies of aging and dementia. The cases group consists of 24 individuals with high levels of β-amyloid and other pathological hallmarks of AD, and the control group consists of 24 individuals who have no or very low β-amyloid or other pathologies.
Study data were provided by the Rush Alzheimer's Disease Center, Rush University Medical Center, Chicago. Data collection was supported through funding by NIA grants P30AG10161 (ROS), R01AG15819 (ROSMAP; genomics and RNAseq), R01AG17917 (MAP), R01AG30146, R01AG36042 (5hC methylation, ATACseq), RC2AG036547 (H3K9Ac), R01AG36836 (RNAseq), R01AG48015 (monocyte RNAseq) RF1AG57473 (single nucleus RNAseq), U01AG32984 (genomic and whole exome sequencing), U01AG46152 (ROSMAP AMP-AD, targeted proteomics), U01AG46161(TMT proteomics), U01AG61356 (whole genome sequencing, targeted proteomics, ROSMAP AMP-AD), the Illinois Department of Public Health (ROSMAP), and the Translational Genomics Research Institute (genomic). Additional phenotypic data can be requested at `www.radc.rush.edu`.

1.1.5 Tabula Muris

We obtained the Tabula Muris dataset with ground truth cell type labels from FigShare (`https://figshare.com/projects/Tabula_Muris_Transcriptomic_characterization_of_20_organs_and_tissues_from_Mus_musculus_at_single_cell_resolution/27733`) for the Version 2 release [5]. This dataset includes mouse single-cell transcriptome data sequenced by two tech-

nologies: microfluidic droplet-based, and fluorescence-activated cell sorting (FACS)-based. A QC cutoff was applied in the original empirical study where only cells with at least 500 genes and 50,000 reads/ 1000 UMI are kept. The droplet subset includes data for 422,803 droplets, 55,656 of which passed the QC cutoff. The FACS subset, denoted as TM (FACS) in the paper, contains data for 53,760 cells, 44,949 of which passed the QC cutoff.

1.1.6 Allen Brain Atlas

We downloaded two brain datasets from Allen Brain Atlas [6] (accessed 03/21/2021). The human primary motor cortex dataset (HumM1C) includes single-nucleus transcriptomes from 76,533 nuclei from the primary motor cortex (M1C) of 2 post-mortem human brain specimens. In total, 127 transcriptomic cell types are present in this dataset. The sample processing follows the 10x Genomics pipeline. To generate the ground-truth cell labels, the default 10x Cell Ranger v3 pipeline was used except substituting the curated genome annotation used for SMART-seq v4 quantification. The mouse brain dataset includes single-cell transcriptomes from more than 20 areas of mouse cortex and the hippocampus, which has 1,093,785 cells in total. Samples were collected from male and female mice around 8 week-old, from pan-neuronal transgenic lines. The cell transcriptomes were sequenced using 10x Genomics. We used the subclass labels in the metadata used as ground truth cell type label in the current study. We removed the cells and nuclei with subclass label outlier. To encourage better transfer from HumM1C, we subset the mouse brain dataset by keeping the 124,953 cells with region_label MOp, and obtain the MusMOp dataset.

1.1.7 Mouse Retina

We obtained the MR dataset from `https://hemberg-lab.github.io/scRNA.seq.datasets/mouse/retina/`, which is a collection of mouse retina scRNA-seq datasets from two independent studies, namely Macosko *et al*. [7] with 44808 samples, and Shekhar *et al*. [8] with 27499 samples. Both subsets were sequenced using Drop-seq. We kept the genes shared by the two subsets, and filtered out the 669 samples labeled as Doublets/Contaminants in the Shekhar batch. The merged dataset contains 71638 cells and 12333 genes.

1.2 Experimental details of other scRNA-seq methods

Neural-network based models, including scVI, scVI-LD, scVAE-GM and scETM typically need at least 5000 gradient updates to converge. When running on small datasets, the total number of gradient updates per epoch may be very small (sometimes as low as 1). In these cases, we increase the number of epochs T to ensure the model goes through at least 12000 gradient updates, i.e. $T = \max(800, 12000\frac{B}{N})$, where N is the number of cells in the dataset and B is the mini-batch size.

1.2.1 Seurat v3

We downloaded Seurat v3 (version 3.1.5) from CRAN [9]. We followed the steps outlined by the integration workflow (`https://satijalab.org/seurat/v3.2/integration.html`) which includes NormalizeData, FindVariableFeatures, FindIntegrationAnchors, and IntegrateData. To make the comparisons more equitable, we set the min.features=0 to avoid exclusion of cells. All other parameters were set as default. We noted that, with batch integration turned on, Seurat reports error in the integration step due to the high number of anchors arising from the 48 individuals (batch variable in AD), which is a known implementation issue with the standard Seurat v3 integration workflow [10]. We therefore turned off the batch integration for AD in the benchmarking experiments (see **Clustering performance benchmark and visualization** and **Efficiency and scalability benchmark of the existing methods** in the main text) and followed the steps described in the Guided Clustering Tutorial (`https://satijalab.org/seurat/v3.2/pbmc3k_tutorial.html`).

1.2.2 Scanorama

We downloaded the source code from GitHub `brianhe/scanorama`. We used the `integrate_scanpy` function for dataset integration and batch correction as suggested by the guided tutorial. All parameters were set as default. The algorithm performs a PCA on the stacked datasets and uses 100 PCs for downstream computation.

1.2.3 Harmony

We downloaded the source code from GitHub `slowkow/harmonypy` suggested by the primary repository `immunogenomics/harmony` and followed the preprocessing (normalization and top variable gene selection) described in the publication and the integration steps in the provided tutorial. We used the `run_harmony` function to obtain the corrected PCA embeddings and used 50 PCs as input. All other parameters were set as default.

1.2.4 LIGER

We used the official implementation provided on the website of the LIGER package. For the convenience of implementation, we followed the usage tutorial using Seurat Wrapper to process the raw data and then ran LIGER with default parameters.

1.2.5 scVI/scVI-LD

We downloaded the implementation from the Github repository `YosefLab/scVI`. We used the default model, which has one layer for both the encoder and decoder (for scVI-LD the decoder is a latent dimensions-by-genes matrix), 128 hidden units, 10 latent dimensions and ZINB distribution for modeling the data. We chose 10^{-3} as the learning rate and trained on each unprocessed dataset for 400 epochs, following the provided tutorials. We change the training batch

size to 2000 for faster training. We obtained the cell embeddings via the `get_latent` method. We also compared the performance of scVI(-LD) with 10 and 100 latent dimensions on the five benchmark scRNA-seq datasets (Supp. Table 19). We found that in most cases, scVI(-LD) with 10 latent dimensions performs better than scVI(-LD) with 100 latent dimensions, justifying the hyperparameter choices made by the scVI(-LD) authors. When evaluating the interpretability aspect of scVI-LD, we use 100 latent dimensions for fair comparison with scETM.

1.2.6 scVAE

We downloaded the implementation from GitHub repository `scvae/scvae`. We set the hidden units to be $(256, 128)$ for the encoder. The decoder is symmetric to the encoder. Latent dimension was set to 128 to match scETM. We chose 10^{-4} as the learning rate and NB distribution for modeling the data following the authors' recommendation. We trained on each unprocessed dataset for 400 epochs with batch size of 250, including a 200-epoch warm-up for the KL divergence loss. In the scalability benchmark, we disabled the time-consuming per-epoch checkpoints to match other methods. The model did not converge on the Human Pancreatic Islet dataset, where the ELBO went to infinity. It failed to extract meaningful information from the Tabula Muris dataset, resulting in an ARI of 0.0.

2 Supplementary Figures

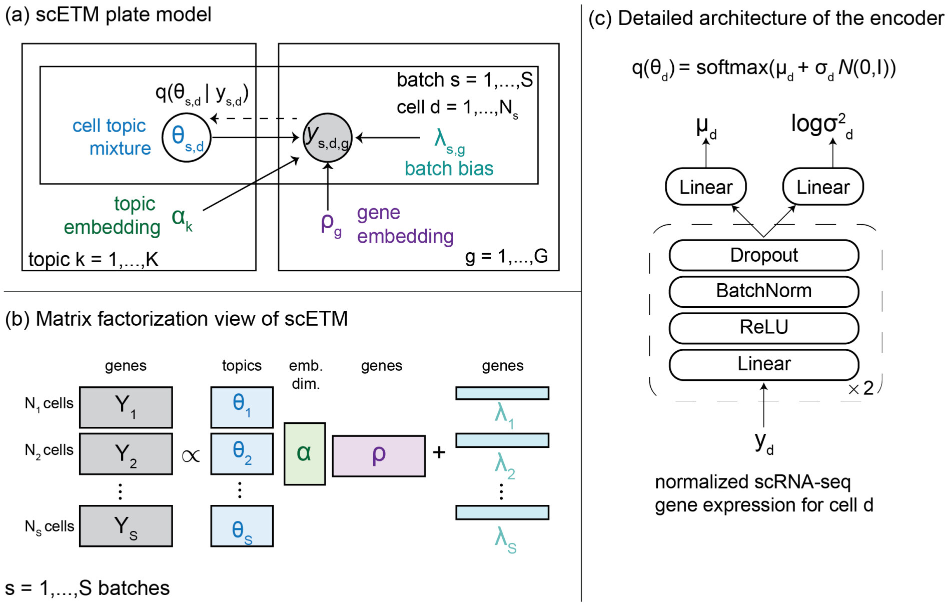

Figure 1: **scETM model details.** (a) The plate model for scETM. We model the scRNA-profile count matrix $y_{d,g}$ in cell d and gene g across S batches by a multinomial distribution with the rate parameterized by cell topic mixture θ, topic embedding α, gene embedding ρ, and batch effects λ. (b) Matrix factorization view of scETM. (c) Encoder architecture for inferring the cell topic mixture θ.

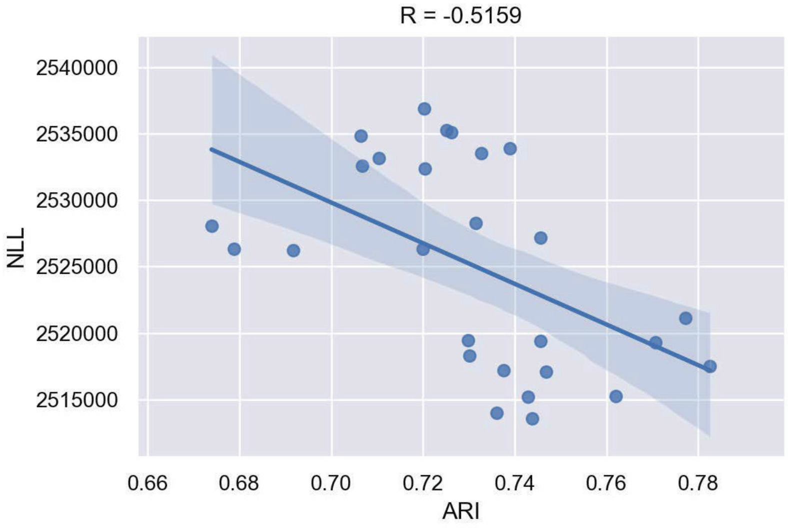

Figure 2: **Relationship between average negative log-likelihood (NLL) and adjusted Rand Index (ARI) on the TM dataset.** Each point denotes the performance of a trained scETM instance with a specific hyperparameter configuration (e.g., encoder hidden size, topic number, embedding dimensions, etc), averaged over three runs with different random seeds. The translucent band is the 95% confidence interval estimated using bootstrap by the built-in `seaborn.regplot` function.

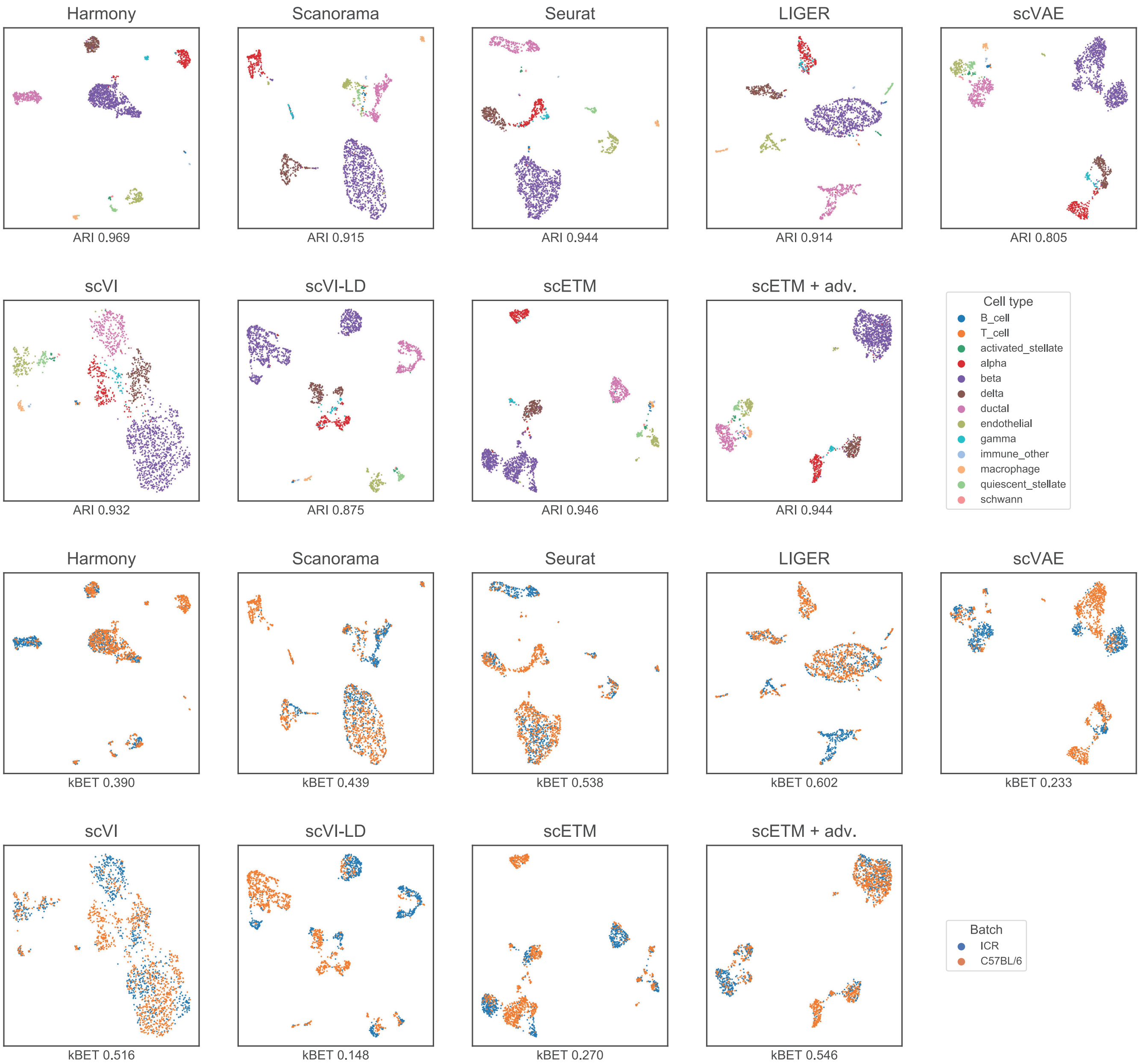

Figure 3: **Integration and batch correction on the Mouse Pancreas (MP) dataset**. Each panel shows the MP cell clusters using UMAP based on the cell embeddings obtained by each of the 9 methods. The cells are colored by cell types in the first two rows and by batches, which are the two mouse strains, in the last two rows.

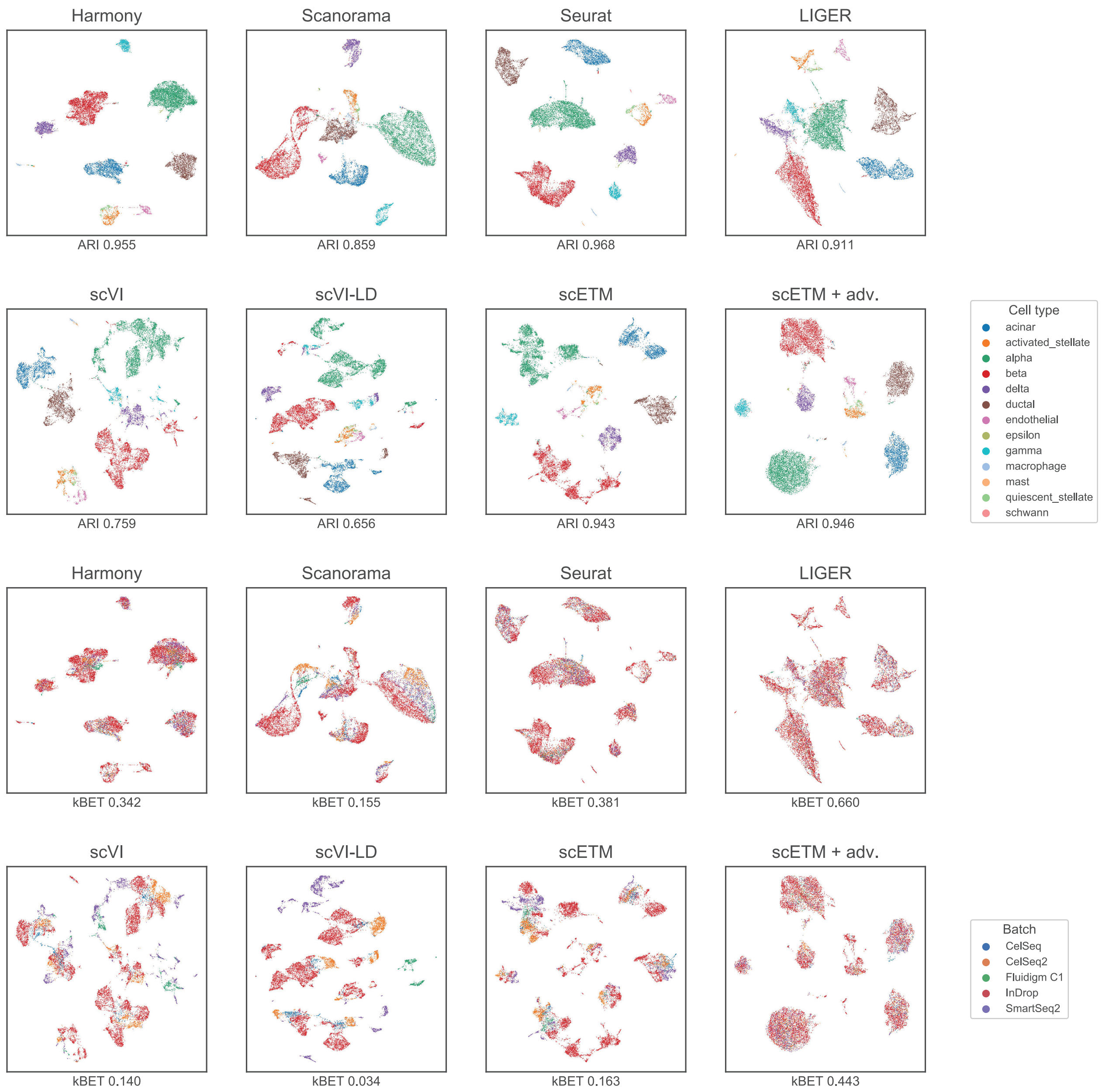

Figure 4: **Integration and batch correction on the Human Pancreas (HP) dataset**. Each panel shows the HP cell clusters using UMAP based on the cell embeddings obtained by each of the 9 methods. The cells are colored by cell types in the first two rows and by batches, which are the five sequencing technologies, in the last two rows.

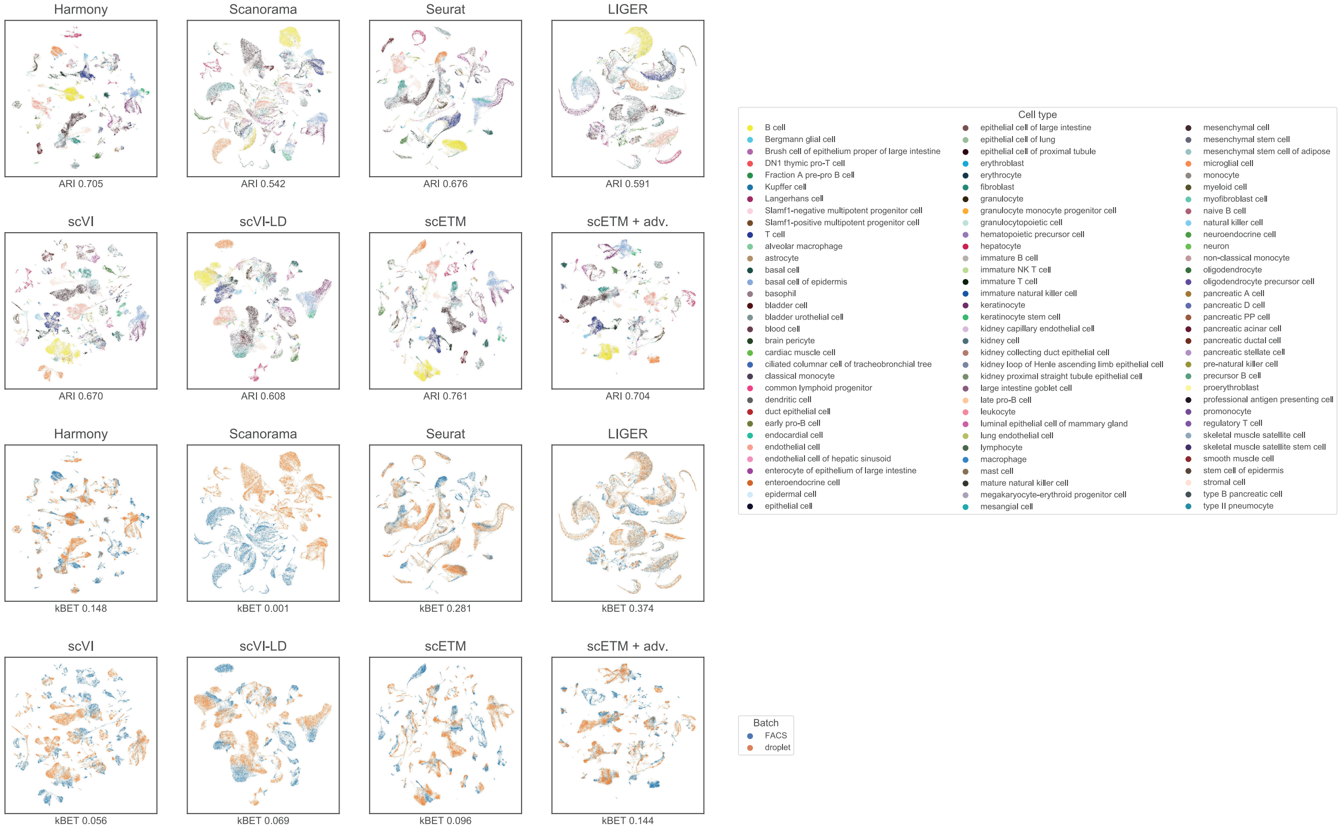

Figure 5: **Integration and batch correction on the Tabula Muris (TM) dataset**. Each panel shows the TM cell clusters using UMAP based on the cell embeddings obtained by each of the 9 methods. The cells are colored by cell types in the first two rows and by batches, which are the two sequencing technologies, in the last two rows.

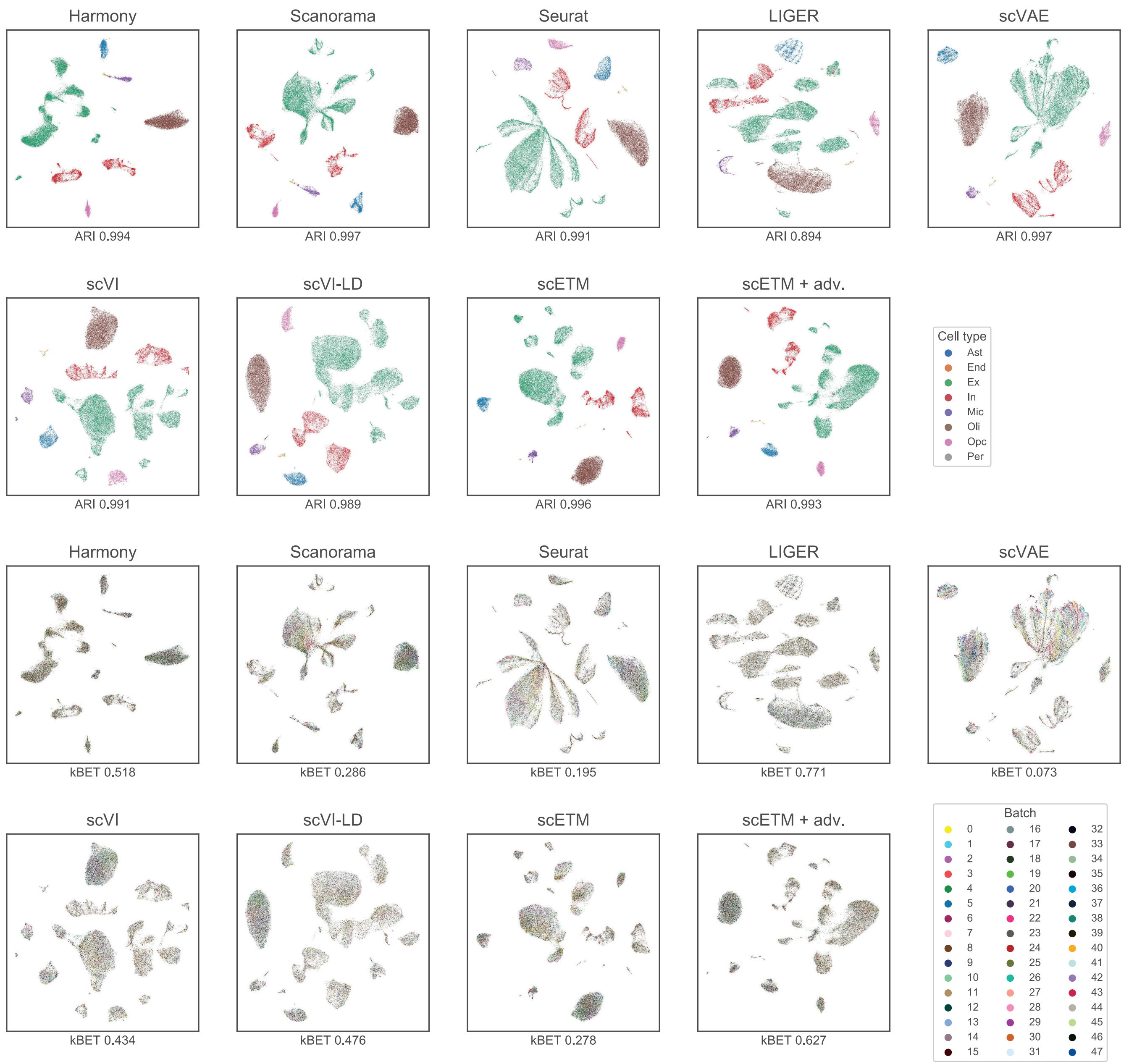

Figure 6: **Integration and batch correction on the Alzheimer's Disease (AD) dataset.** Each panel shows the prefrontal cortex (PFC) cell clusters using UMAP based on the cell embeddings obtained by each of the 9 methods. The cells are colored by cell types in the first two rows and by batches, which are the 48 donors, in the last two rows.

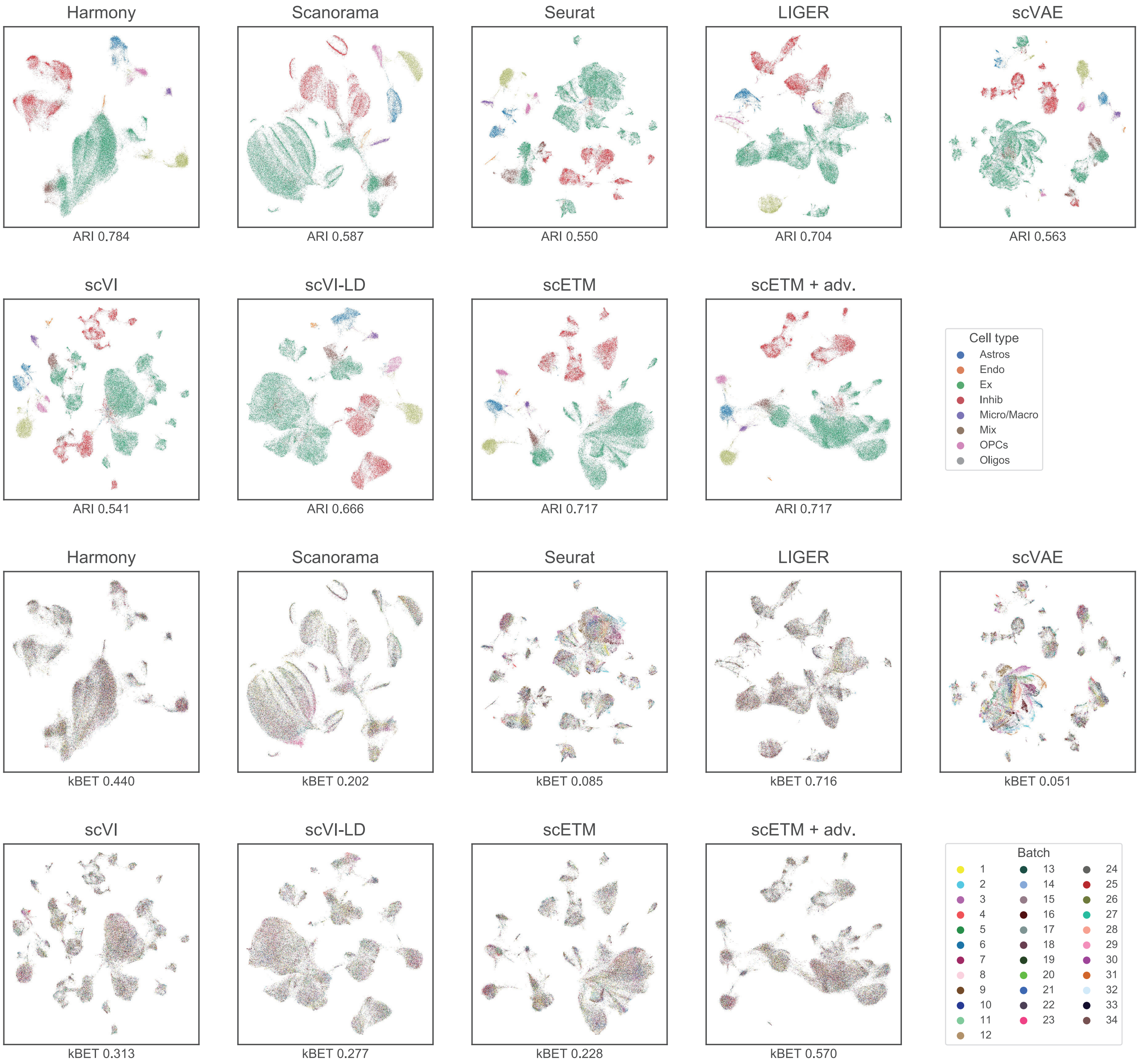

Figure 7: **Integration and batch correction on the Major Depressive Disorder (MDD) dataset**. Each panel shows the prefrontal cortex (PFC) cell clusters from the MDD or healthy subjects using UMAP based on the cell embeddings obtained by each of the 9 methods. The cells are colored by cell types in the first two rows and by batches, which are the 34 donors, in the last two rows.

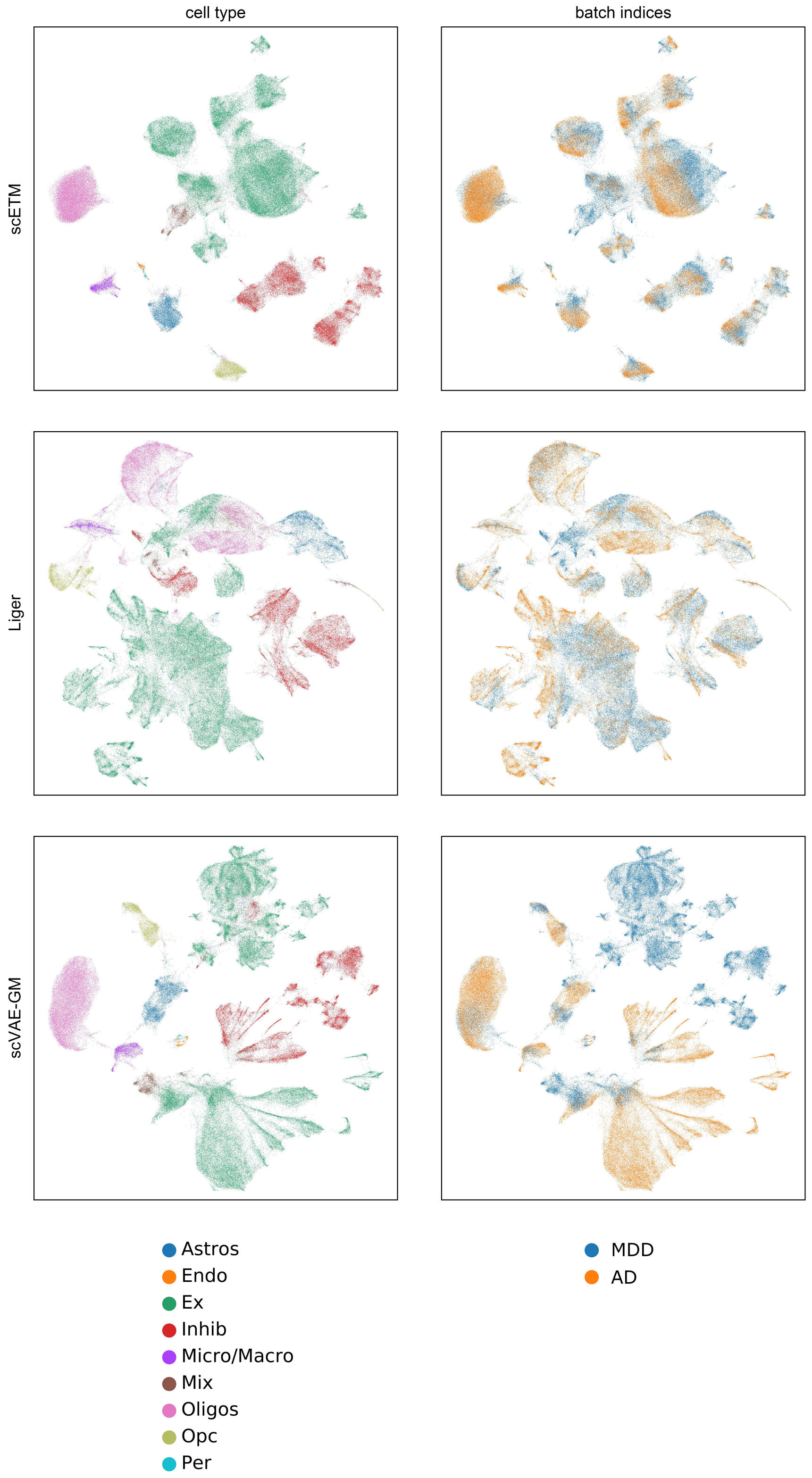

Figure 8: UMAP visualization of scETM, LIGER and scVAE-GM cell embeddings on the combined MDD and AD benchmark dataset, which includes in total 148247 cells and 3000 genes.

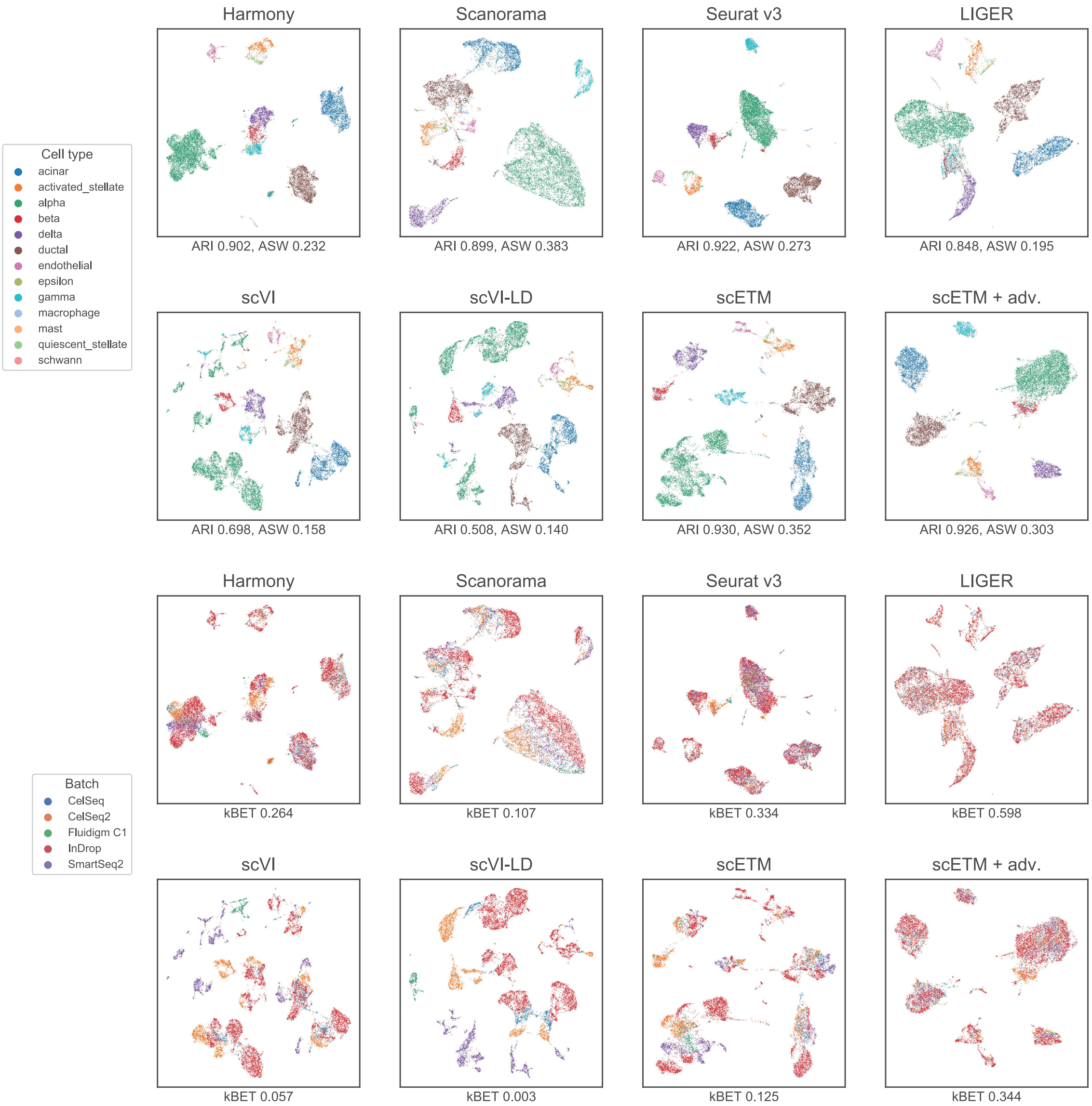

Figure 9: **Integration and batch correction on the HP–beta dataset.** Each panel shows the HP–beta cell clusters using UMAP based on the cell embeddings obtained by each of the 8 methods (scVAE did not converge on this dataset). The cells are colored by cell types in the first two rows and by batches, which are the five sequencing technologies, in the last two rows. The ARI and kBET scores of each method are shown below each plot.

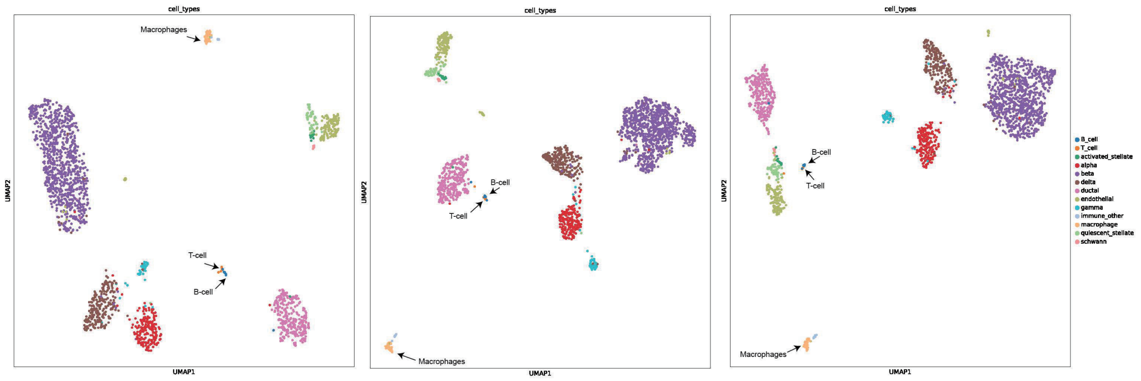

Figure 10: **Reproducibility of the separation of T-cells and B-cells from macrophages.** We trained scETM on TM (FACS) and applied it to the MP data. To assess the reproducibility, we repeated the experiments 3 times using different random seeds to initialize the model.

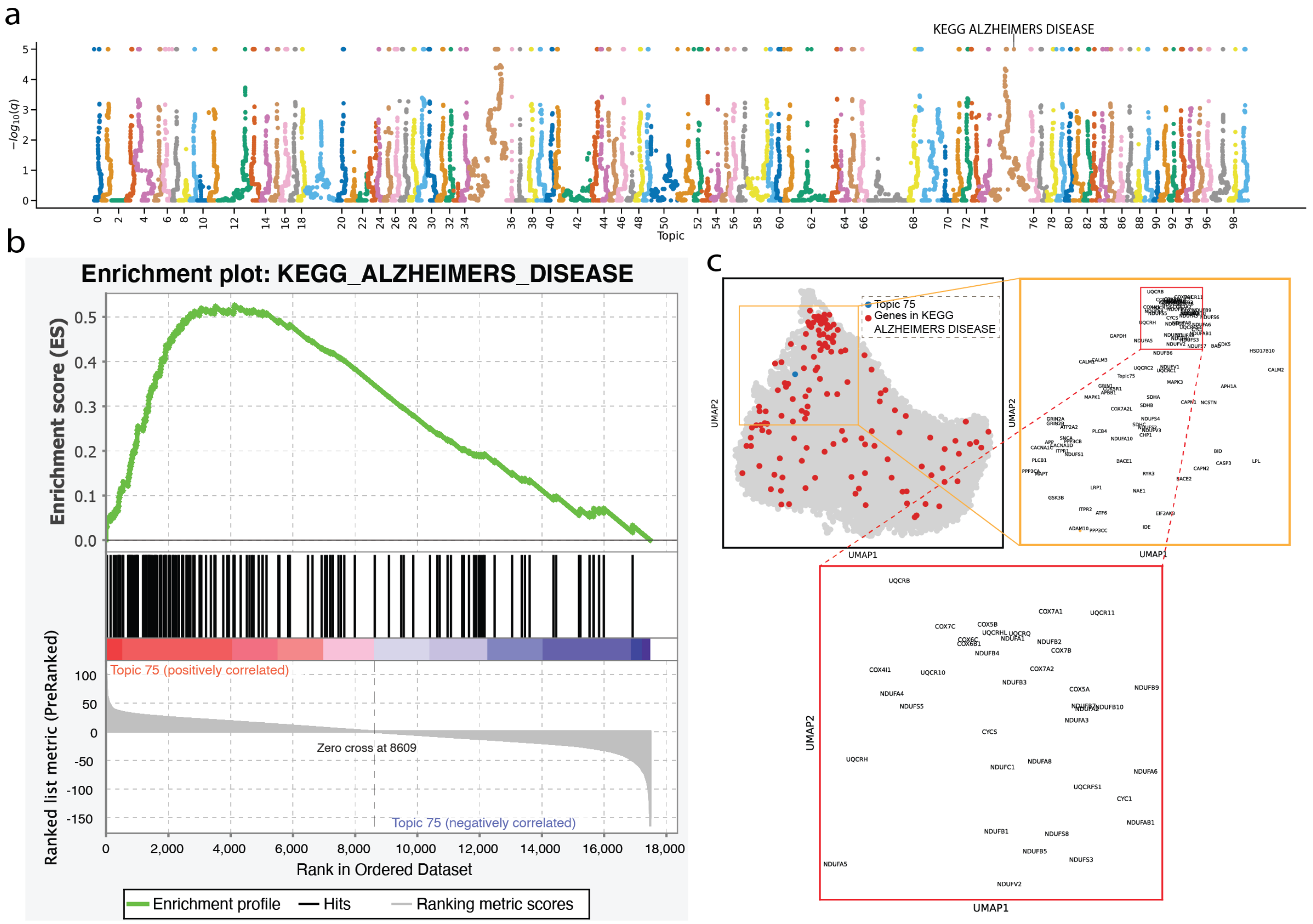

Figure 11: **Alzheimer's Disease dataset gene and topic embedding. (a)** Manhattan plot of the GSEA results on the 100 scETM topic learned from the AD dataset. The maximum -log q-value is capped at 5. **(b)** Leading edge analysis of KEGG_ALZHEIMERS_DISEASE (KAD) pathway. The genes are ranked by the topic score (i.e., the unnormalized topic mixture beta) under Topic 75. Topic 75 is significantly enriched in KAD pathway (GSEA permutation test q-value=0). **(c)** UMAP visualization of the embeddings of all genes in AD and Topic 75. A magnified view of the cluster shows the KAD pathway genes that are near Topic 75.

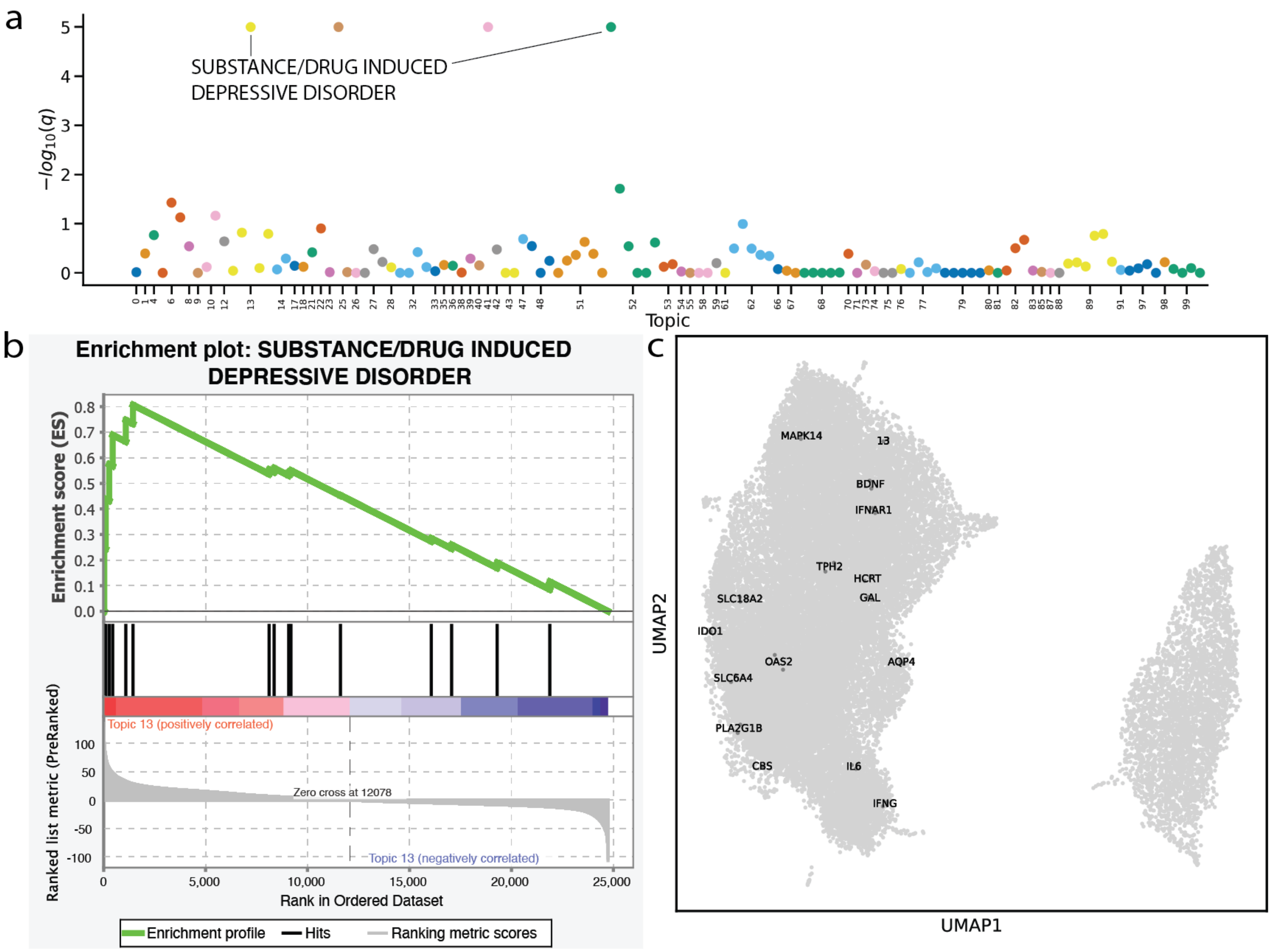

Figure 12: **MDD dataset gene and topic embedding. (a)** Manhattan plot of the GSEA results on the 100 scETM topic learned from the MDD dataset. **(b)** Leading edge analysis of SUBSTANCE/DRUG INDUCED DEPRESSIVE DISORDER (SID) pathway. Topic 13 is significantly enriched in SID pathway (GSEA permutation test q-value=0). **(c)** UMAP visualization of the embeddings of all genes in the MDD dataset and Topic 13. A magnified view of the cluster shows the SID pathway genes that are near Topic 13.

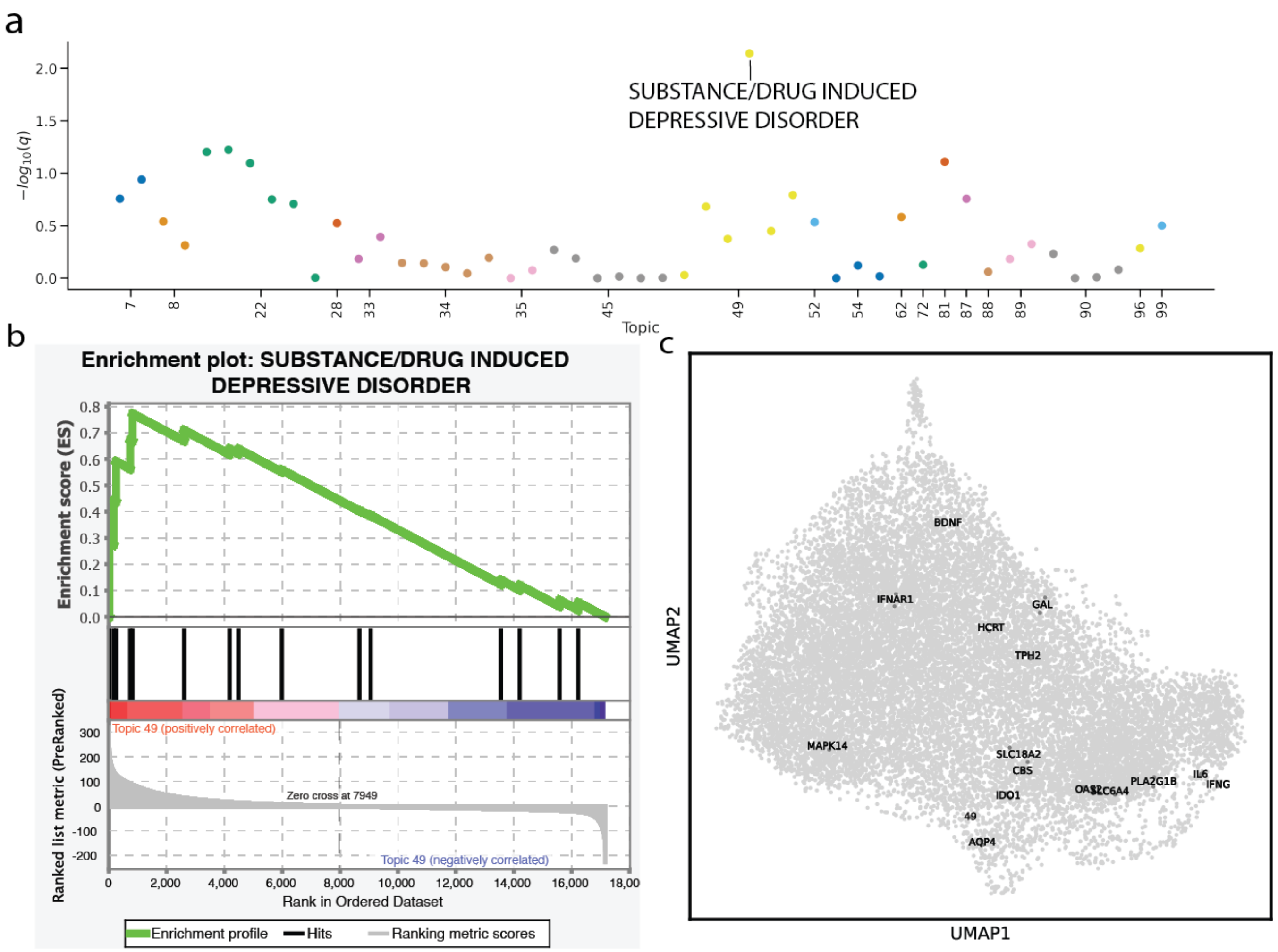

Figure 13: **Gene and topic embedding of MDD (coding genes only). (a)** Manhattan plot of the GSEA results on the 100 scETM topic learned from the MDD dataset. **(b)** Leading edge analysis of SUBSTANCE/DRUG INDUCED DEPRESSIVE DISORDER (SID) pathway. Topic 49 is significantly enriched in SID pathway (GSEA permutation test q-value=0). **(c)** UMAP visualization of the embeddings of all protein coding genes in MDD dataset and Topic 49. A magnified view of the cluster shows the SID pathway genes that are near Topic 49.

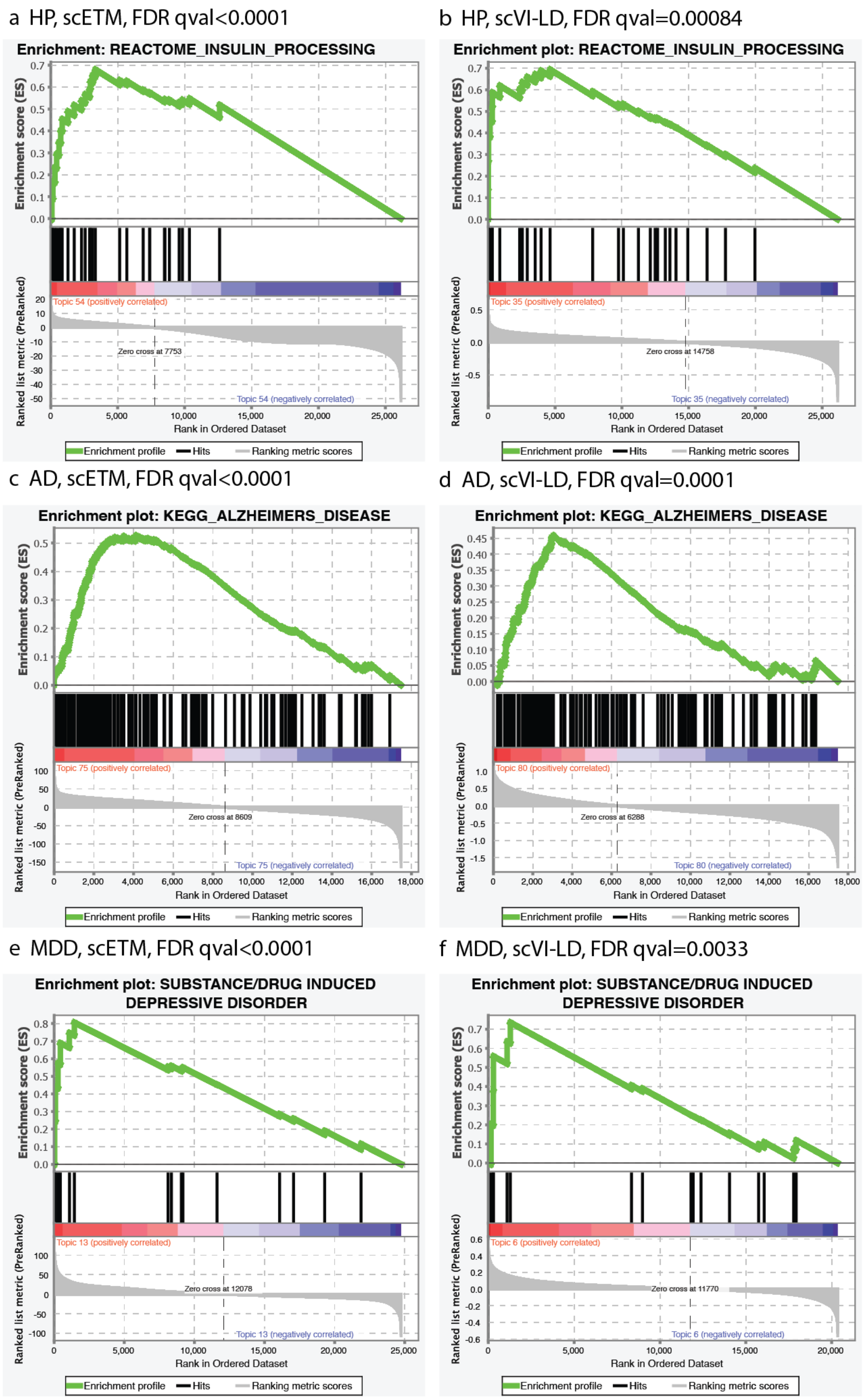

Figure 14: **GSEA leading edge plots for scETM (left column) and scVI-LD (right column) on (a,b) Human Pancreas (HP), (c,d) Alzheimer's Disease (AD), and (e,f) Major Depressive Disorder (MDD).** Due to the disease relevance, we showed Reactome Insulin Processing, KEGG Alzheimer's Disease, and PsyGeNet substance/drug induced depressive disorder for HP, AD, and MDD, respectively.

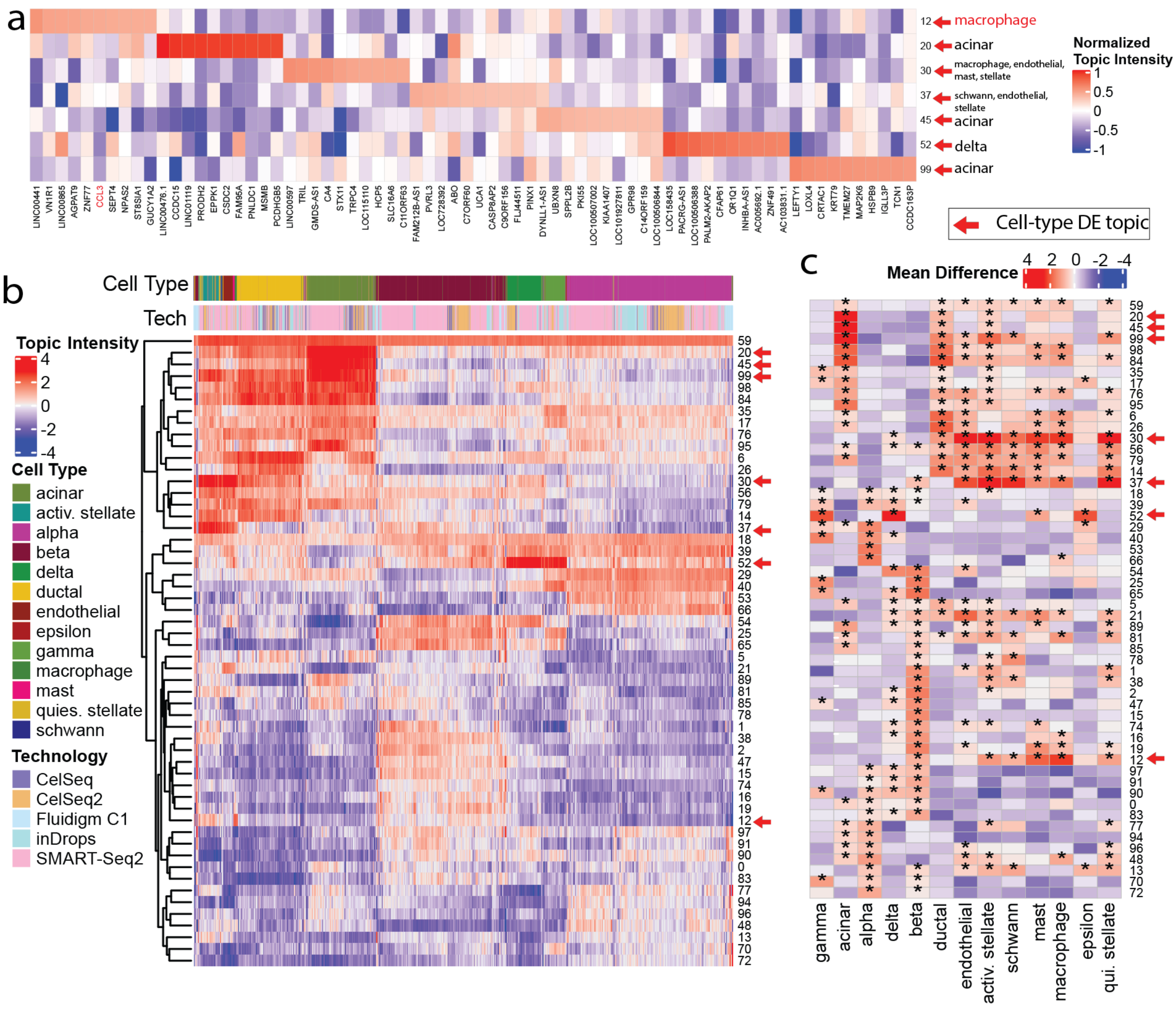

Figure 15: **scETM topic embeddings of Human Pancreas scRNA-seq data. (a)** Gene-topics heatmap of top 10 genes in each topic based on topic intensity. The top genes which are known as cell-type marker genes based on PanglaoDB are highlighted (rows). For visualization purposes, we divided the topic values by the maximum absolute value within the same topic. Only cell-type and disease differntial topics are shown. **(b)** Topic intensity of cells (n=10,000) sub-sampled from the HP dataset. Topic intensities shown here are the Gaussian mean before applying softmax. Only the select topics with the sum of absolute values greater than 1500 across all sampled cells are shown. The two color bars show cell types and batch identifiers (i.e., sequencing technologies). **(c)** Differential expression analysis of topics across the 13 cell types. Colors indicate mean differences between cell groups with and without a certain label (cell-type). Asterisks indicate Bonferroni-corrected empirical p-value < 0.05 for one-sided permutation tests of up-regulated topics in each cell-type labels. The number of permutations in each test is 100,000.

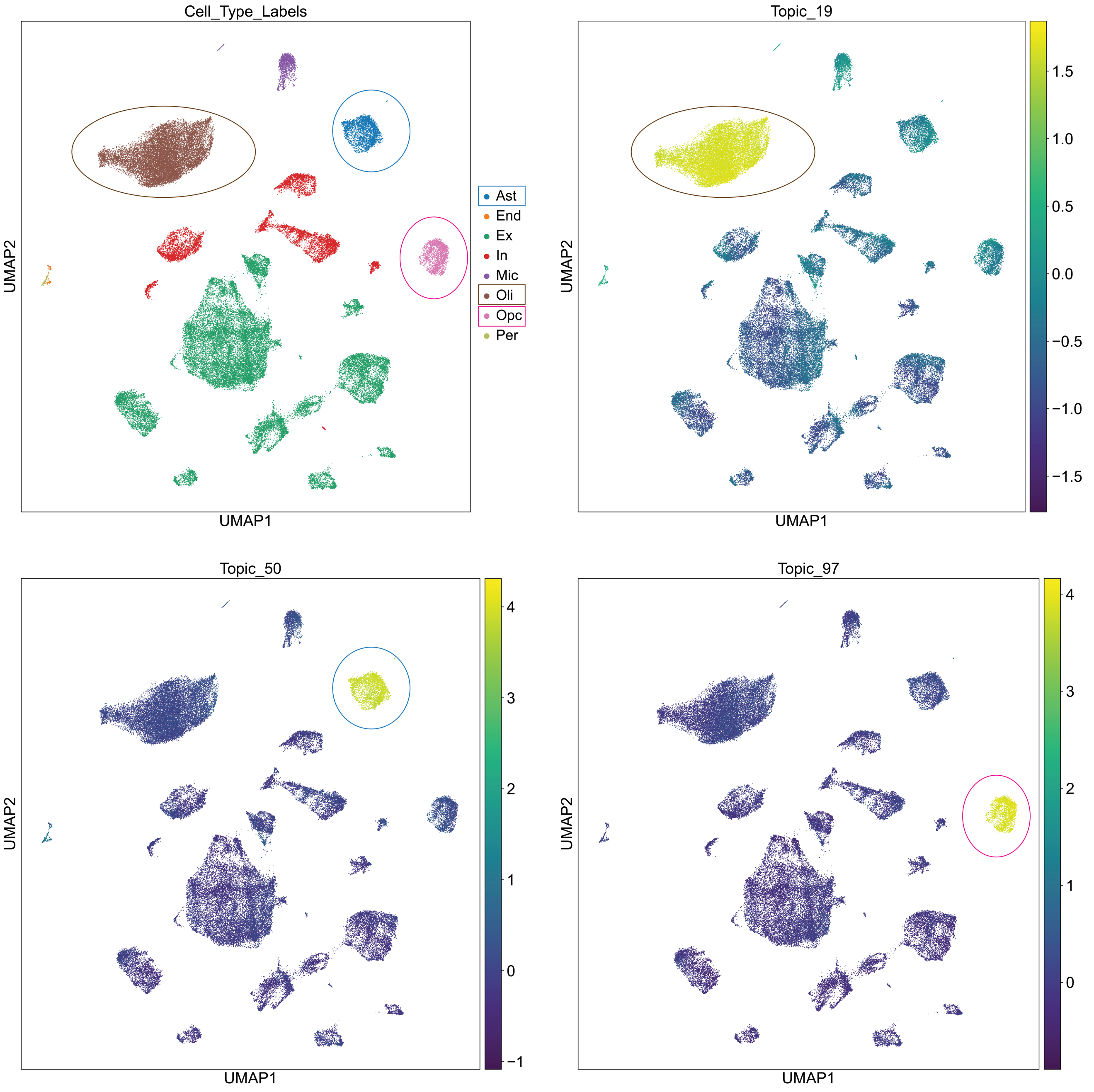

Figure 16: UMAP cell embedding visualization on the AD dataset, colored by differentially expressed topics (or metagenes) and ground truth labels for the cell types. Circled cell clusters has been discussed in the main text (see **Differential scETM topics in disease conditions and cell types** section).

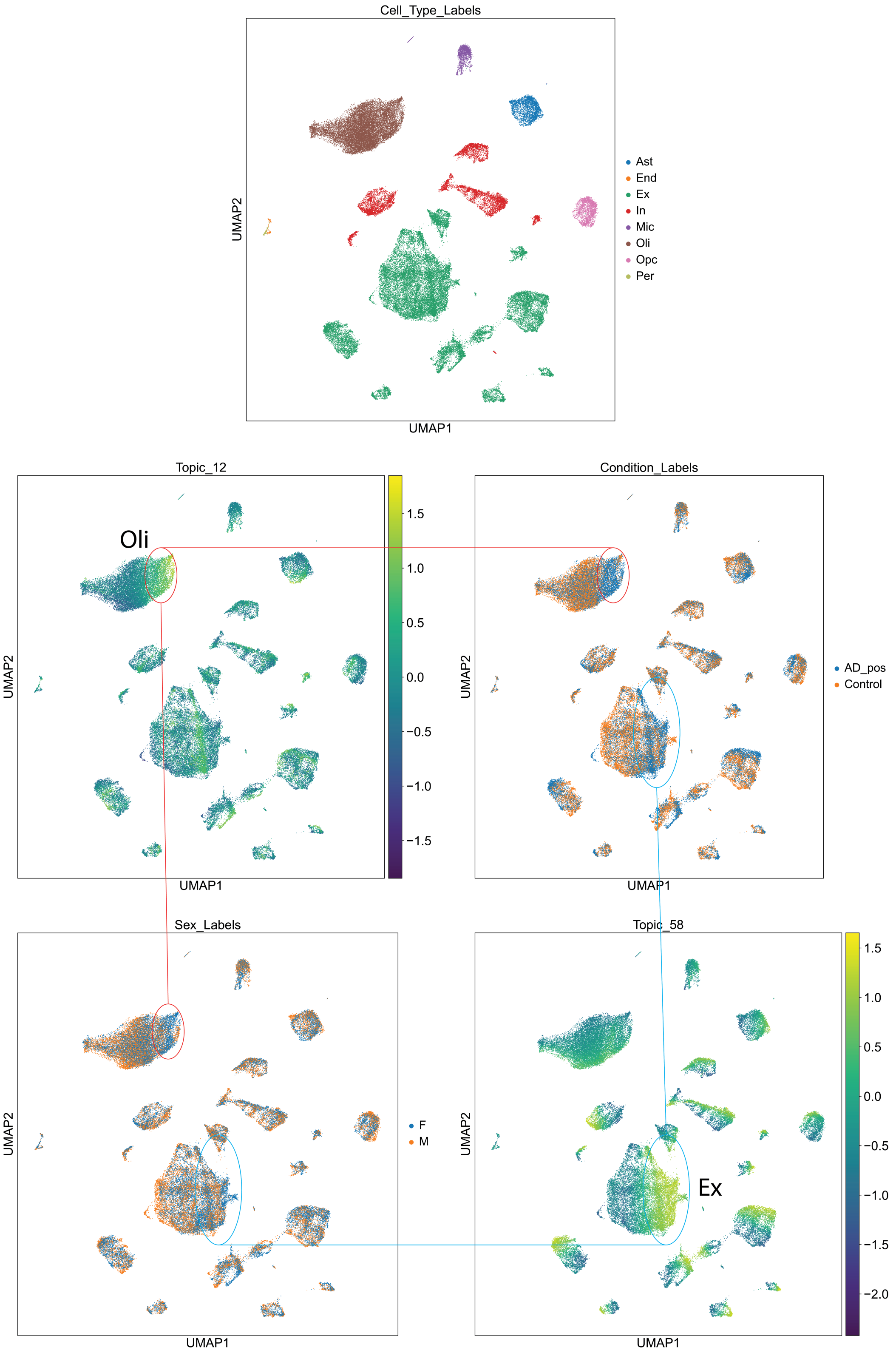

Figure 17: UMAP cell embedding visualization on the AD dataset, colored by differentially expressed topics (or metagenes) and AD/control or Male/Female labels. Circled cell clusters were discussed in the main text (see **Differential scETM topics in disease conditions and cell types** section).

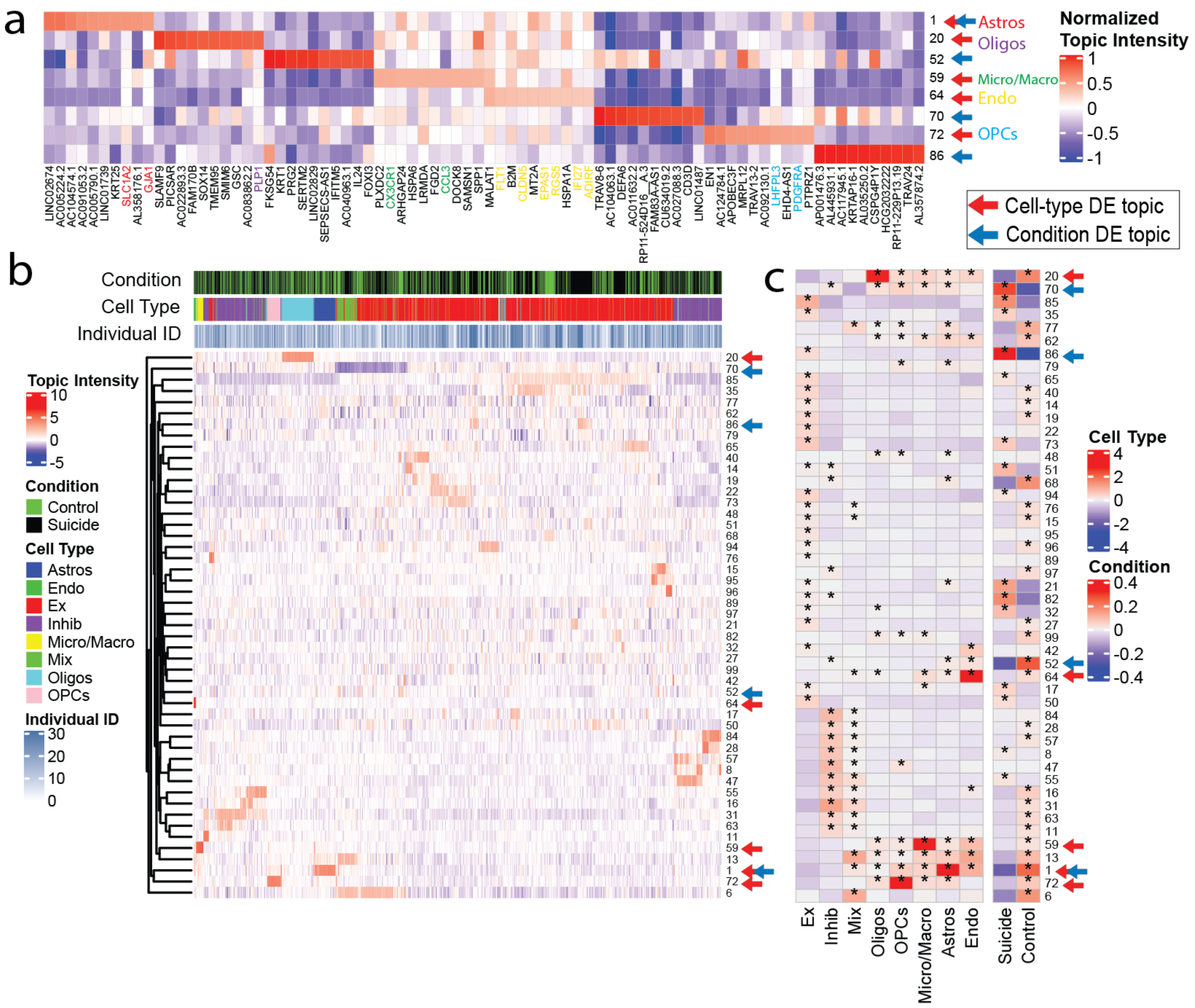

Figure 18: **scETM-topic embeddings learned from the Major Depressive Disorder scRNA-seq data. (a)** Gene-topics heatmap of top 10 genes in each topic based on topic intensity. The top genes which are known as cell-type marker genes based on PanglaoDB are highlighted (rows). For visualization purposes, we divided the topic values by the maximum absolute value within the same topic. Only cell-type and disease differential topics are shown. **(b)** Topics intensity of cells (n=10,000) sub-sampled from the MDD dataset. Topic intensities shown here are the Gaussian mean before applying softmax. Only the select topics with the sum of absolute values greater than 1500 across all sampled cells are shown. The three color bars show disease conditions, cell types, and batch identifiers (i.e., subject IDs). **(c)** Differential expression analysis of topics across the 8 cell types and 2 clinical conditions. Colors indicate mean differences between cell groups with and without a certain label (cell-type or condition). Asterisks indicate Bonferroni-corrected empirical p-value < 0.05 for one-sided permutation tests of up-regulated topics in each cell-type and disease labels. The number of permutations in each test is 100,000.

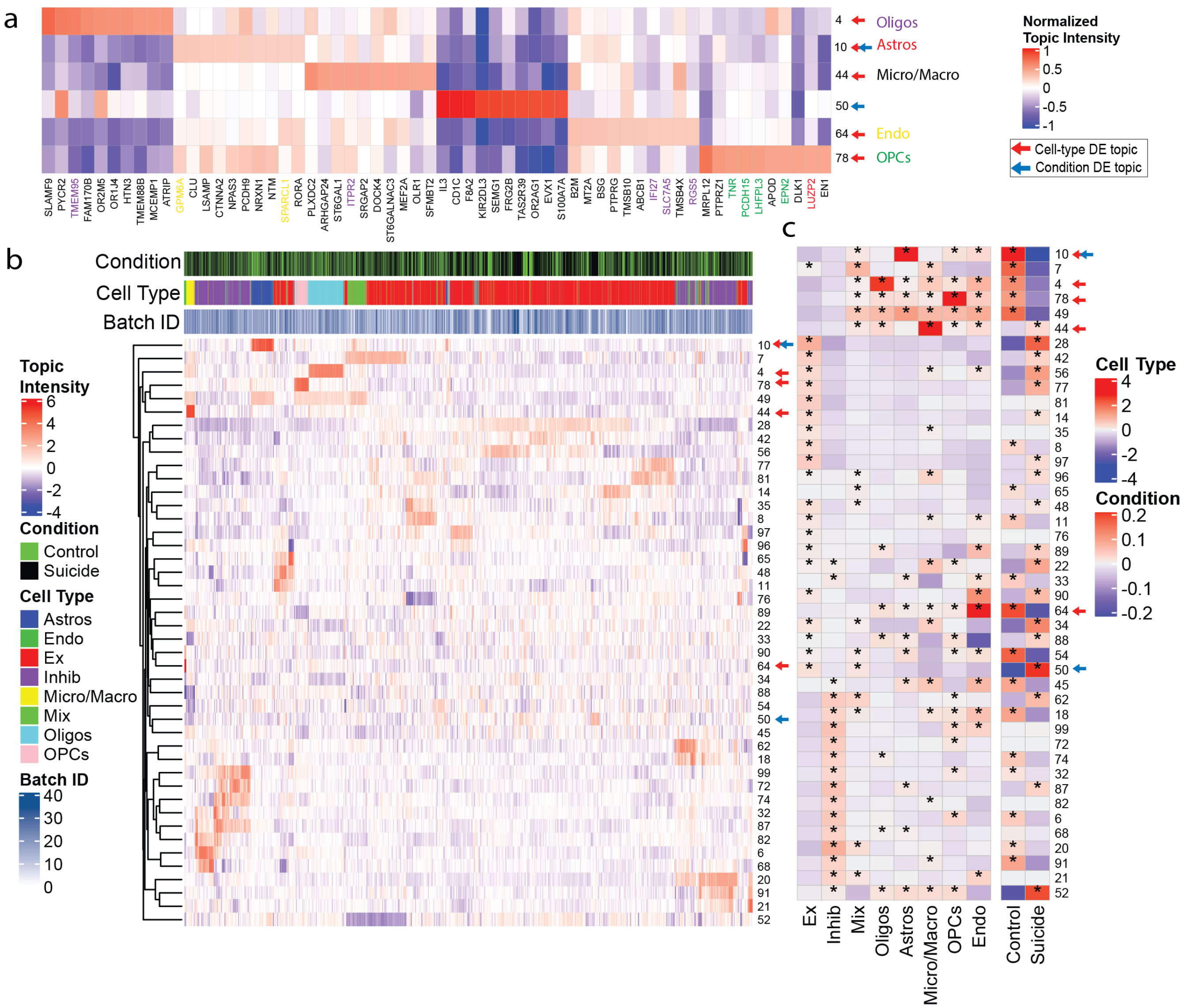

Figure 19: **scETM-topic embeddings learned from the Major Depressive Disorder scRNA-seq data with genes restricted to protein coding genes. (a)** Gene-topics heatmap of top 10 genes in each topic based on topic intensity. **(b)** Topics intensity of cells (n=10,000) sub-sampled from the MDD - coding genes only dataset. **(c)** Differential expression analysis of topics across the 8 cell types and 2 clinical conditions.

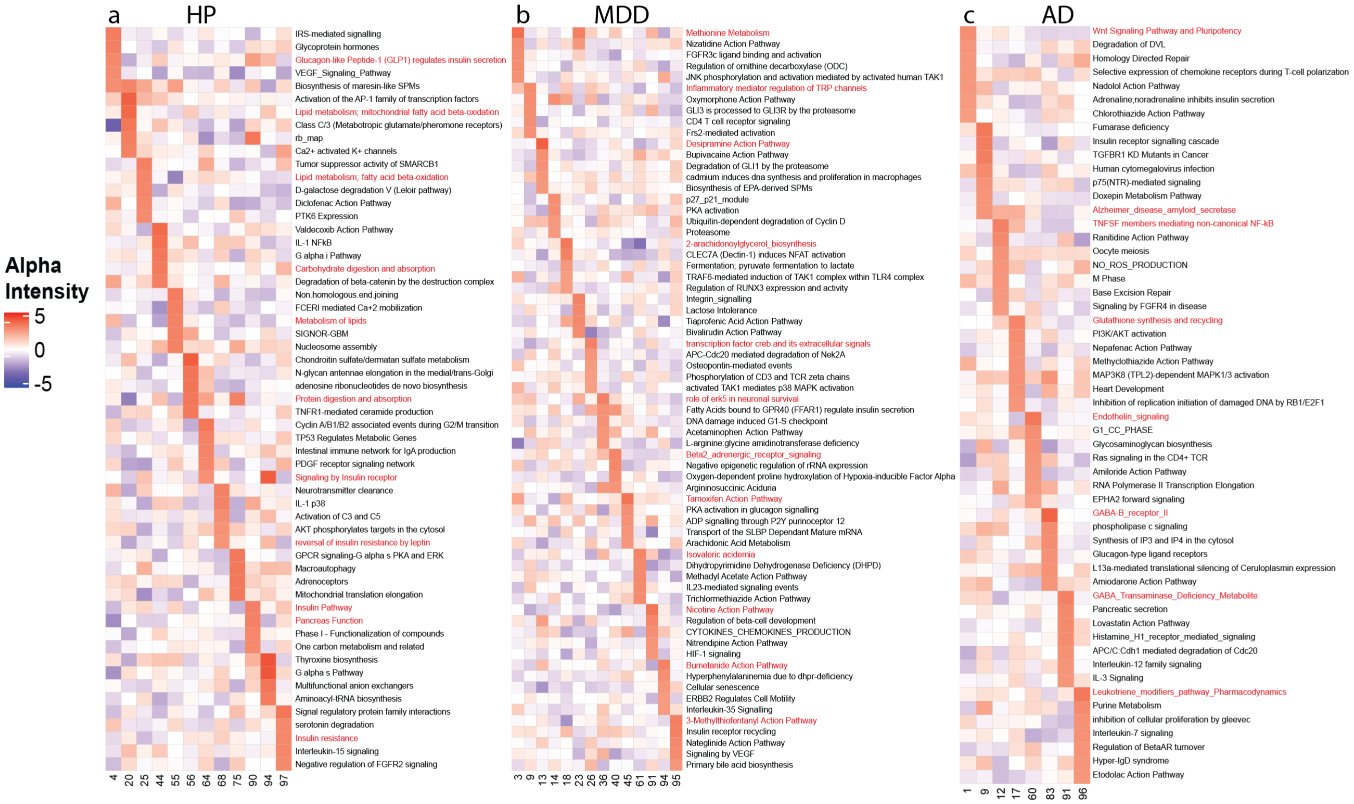

Figure 20: **p-scETM pathway-topics embeddings by fixing pathDIP gene set database as ρ. (a)** The pathway-topics heatmap of top 5 pathways in selected topics, inferred by a p-scETM model trained on HP. Pathways potentially related to pancreas function, insulin signalling and digestion are highlighted. **(b)** The pathway-topics heatmap of top 5 pathways in selected topics, inferred by a p-scETM model trained on MDD. Pathways potentially related to MDD pathogenesis and therapeutic targets are highlighted. **(c)** The pathway-topics heatmap of top 7 pathways in selected topics, inferred by a p-scETM model trained on AD. Pathways potentially related to AD pathogenesis and therapeutic targets are highlighted.

3 Supplementary Tables

	MP	HP	TM	AD	MDD	MR
Harmony	0.928	0.927	0.827	0.983	0.705	0.726
Scanorama	0.845	0.816	0.766	0.988	0.561	0.747
Seurat	0.913	0.947	0.825	0.967	0.540	0.783
scVAE-GM	0.764	NA	NA	0.989	0.526	0.781
scVI	0.876	0.783	0.835	0.977	0.534	0.763
LIGER	0.875	0.876	0.757	0.861	0.618	0.742
scVI-LD	0.831	0.713	0.797	0.975	0.614	0.765
scETM+adv	0.852	0.908	0.838	0.985	0.598	0.747
scETM	0.902	0.902	0.856	0.987	0.583	0.809
scETM$-\lambda$	0.819	0.644	0.819	0.987	0.604	0.764
Batch Effect	Strain	Tech.	Tech.	Ind.	Ind.	Studies

Table 1: Normalized Mutual Information (NMI) between ground truth cell types and leiden clusters on 6 benchmark scRNA-seq datasets. NA is reported for models that did not converge. scETM performances with or without the linear batch correction (scETM, scETM$-\lambda$) are both reported. scETM+adv is scETM plus adversarial network loss to further correct batch effects. Batch variables include strain, sequencing technologies ("Tech.") and individuals ("Ind."). NA is reported for models that did not converge. More details are described in Table 1 caption and Methods in the main text.

	MP		HP	
	ARI	NLL	ARI	NLL
Current model	0.951	6.721	0.943	7.155
Encoder arch (256, 128)	0.897	6.741	0.940	7.151
Encoder arch (512, 256, 128)	0.898	6.722	0.936	7.159
Gene emb. dim. 25	0.920	6.716	0.945	7.166
Gene emb. dim. 100	0.916	6.734	0.944	7.155
10 topics	0.941	6.723	0.940	7.168
200 topics	0.915	6.771	0.944	7.152

Table 2: Robustness analysis of the scETM model. Changing the encoder architecture, gene embedding dimensions and number of topics has limited impact on model performance. We report the average ARI and the held-out negative log-likehood (NLL) of three runs with different random seeds. The current model has an encoder with one 128-dim hidden layer, a gene embedding dimension of 400 and 50 topics.

	MP		HP	
	ARI	NLL	ARI	NLL
Current model	0.951	6.721	0.943	7.155
- BatchNorm	0.844	6.724	0.834	7.149
- Total count Normalization	0.726	6.815×10^4	0.388	2.041×10^6
- BatchNorm - Total count Normalization	0.000	6.420×10^4	0.000	1.909×10^6
- Batch correction module	0.851	6.724	0.471	7.163

Table 3: Ablation study of the scETM model. We report the average ARI of three repeated trails.

	CelSeq	CelSeq2	Fludigm C1	InDrop	SmartSeq2
acinar	229	274	21	1152	188
activated stellate	19	90	16	294	55
alpha	213	844	241	2309	1008
**beta**	0	445	0	0	0
delta	50	203	25	608	127
ductal	304	257	34	915	444
endothelial	5	21	14	235	21
epsilon	1	4	1	16	8
gamma	18	110	18	266	213
macrophage	1	15	1	55	7
mast	1	6	3	39	7
quiescent stellate	1	12	1	160	6
schwann	1	4	5	13	2

Table 4: Cell type distribution of HP–beta. Beta cells (boldfaced) are removed from all batches except CelSeq2 of the HP dataset.

	ARI	ASW	ASW-beta	kBET
Seurat v3	0.9225	0.2732	0.1490	0.3337
Harmony	0.9024	0.2323	0.1558	0.2637
Scanorama	0.8989	0.3832	0.3203	0.1067
LIGER	0.8476	0.1946	0.0912	0.5978
scVI-LD	0.5077	0.1397	0.6135	0.0031
scVI	0.6975	0.1579	0.4044	0.0567
scETM+adv	0.9265	0.3026	0.0045	0.3445
scETM	0.9298	0.3525	0.5370	0.1247

Table 5: Batch overcorrection analysis on HP–beta. We report ARI, ASW, ASW-beta (SWs averaged over all beta cells) and kBET scores on the HP–beta dataset.

	Shekhar *et al.*	Macosko *et al.*
amacrine	252	4426
astrocytes	0	54
bipolar	23494	6285
cones	48	1868
fibroblasts	0	85
ganglion	0	432
horizontal	0	252
microglia	0	67
muller	2945	1624
pericytes	0	63
rods	91	29400
vascular endothelium	0	252

Table 6: Cell type distribution of MR.

	ARI	ASW	kBET
Harmony	0.7632	0.1366	0.0631
Scanorama	0.7795	0.0934	0.0625
Seurat v3	0.7813	0.3464	0.0534
LIGER	0.7135	0.2215	0.1761
scVI	0.7827	0.1331	0.0732
scVI-LD	0.7182	0.1964	0.0217
scETM+adv	0.7720	0.2669	0.1410
scETM	0.8593	0.2873	0.0656

Table 7: Batch overcorrection analysis on MR. We report ARI, ASW and kBET scores on the MR dataset.

Dataset	w/ filtering		w/o filtering	
Metric	ARI	kBET	ARI	kBET
Harmony	0.763	0.063	0.763	0.044
Scanorama	0.780	0.063	0.770	0.052
Seurat v3	0.781	0.053	0.638	0.007
scVI	0.783	0.073	0.774	0.060
LIGER	0.714	0.176	0.463	0.196
scVI-LD	0.718	0.022	0.701	0.013
scETM+adv	0.772	0.141	0.767	0.129
scETM	0.859	0.066	0.854	0.057

Table 8: The impacts of doublets/contaminants on the model performance. ARI and kBET scores of each method, with and without filtering of the 669 doublets/contaminants in the Mouse Retina (MR) dataset, are reported.

Source dataset	MP	TM (FACS)	TM (FACS) w/o Pancreas
Target dataset	MP	MP	MP
ARI	0.9457	0.9409	0.8072
kBET	0.2697	0.3388	0.4772
RCD(B-cell, macrophage)	0.2207	0.7214	0.6686
RCD(macrophage, B-cell)	0.6898	0.8620	0.8368
RCD(T-cell, macrophage)	0.3561	0.7063	0.7002
RCD(macrophage, T-cell)	0.7635	0.8654	0.8413
RCD(B-cell, T-cell)	0.3243	0.4612	0.4307
RCD(T-cell, B-cell)	0.2814	0.4178	0.4698

Table 9: Separation of immune cell types in MP with transfer learning on TM (FACS). We separately trained scETM on MP, TM (FACS) without Pancreas and TM (FACS), and then applied them to the MP dataset to cluster MP cells. We reported the Relative Cluster Distance (RCD) between different immune cell types (T-cell and B-cell) in MP, as well as the overall ARI and kBET scores. RCD is inspired by silhouette width and is defined by $\mathrm{RCD}(A, B) = \frac{d_{AA} - d_{AB}}{\max(d_{AA}, d_{AB})}$, where A, B are two cell types and d is the cosine distance. Higher values indicate cell types A and B are more separated in the embedding. All results are averages over three runs with different random seeds.

Type of transfer	Cross-tissue			Cross-species			
Source dataset	MP	TM (FACS)	TM (FACS) w/o Panc	MP	HP (InDrop)	MusMOp	HumM1C
Target dataset	TM (FACS)	MP	MP	HP (InDrop)	MP	HumM1C	MusMOp
scVI	0.5075	0.4844	0.4197	0.5236	0.4251	0.0900	0.0252
scVI-LD	0.5159	0.3985	0.4317	0.6902	0.4757	0.0895	0.0375
scETM	0.5659	0.9409	0.8072	0.8680	0.7998	0.7105	0.3515

Table 10: Clustering performance on target datasets in the 6 cross-tissue and cross-species transfer learning tasks. The clustering performance is measured by Adjusted Rand Index (ARI) between ground truth cell types and Leiden [11] clusters. The cell clusters based on the cell embedding produced by each method are also depicted in the UMAP visualization in Fig. 4. Abbreviations: MP: Mouse Pancreas; HP (InDrop): Human Pancreas sequenced with InDrop technology; TM (FACS) : A subset of Tabula Muris sequenced with Fluorescence-Activated Single Cell Sorting; TM (FACS) w/o Panc: TM-FACS without Pancreas; HumM1C: human primary motor cortex (from Allen Brain map); MusMOp: Mouse primary motor area (from Allen Brain map).

Type of transfer	Cross-tissue			Cross-species			
Source dataset	MP	TM (FACS)	TM (FACS) w/o Panc	MP	HP (InDrop)	MusMOp	HumM1C
Target dataset	TM (FACS)	MP	MP	HP (InDrop)	MP	HumM1C	MusMOp
scVI	0.0874	0.2570	0.2672	0.1707	0.2276	0.9002	0.8478
scVI-LD	0.0559	0.2564	0.2948	0.1425	0.2204	0.9181	0.8524
scETM	0.0585	0.3388	0.4772	0.1782	0.2930	0.7391	0.7775

Table 11: kBET scores on target datasets during cross-tissue and cross-species transfer learning. Abbreviations are the same as in Supp. Table 10

Pathway Name	scETM	scVI-LD
REACTOME_INSULIN_PROCESSING	15	2
REACTOME_INSULIN_RECEPTOR_RECYCLING	6	0
PID_INSULIN_GLUCOSE_PATHWAY	4	0
WP_PANCREATIC_ADENOCARCINOMA_PATHWAY	2	0
KEGG_PANCREATIC_CANCER	2	0
PID_INSULIN_PATHWAY	2	0
SIG_INSULIN_RECEPTOR_PATHWAY_IN_CARDIAC_MYOCYTES	1	0
REACTOME_REGULATION_OF_GENE_EXPRESSION_IN_LATE_STAGE...	1	0
REACTOME_REGULATION_OF_INSULIN_SECRETION	1	1
REACTOME_REGULATION_OF_INSULIN_LIKE_GROWTH_FACTOR...	1	3
WP_INSULIN_SIGNALING	1	0
BIOCARTA_INSULIN_PATHWAY	1	0
REACTOME_INSULIN_RECEPTOR_SIGNALLING_CASCADE	0	1
REACTOME_SYNTHESIS_SECRETION_AND_INACTIVATION_OF_GLUCOSE...	0	1
REACTOME_SIGNALING_BY_TYPE_1_INSULIN_LIKE_GROWTH_FACTOR...	0	1
WP_FACTORS_AND_PATHWAYS_AFFECTING_INSULINLIKE_GROWTH_FACTOR...	0	1
Total number of pathways	12	7

Table 12: Pathway enrichment statistics for Human Pancreas dataset. Pathways whose names include the keywords "insulin" or "pancreatic" are shown. For each pathway, the number of topics with significant enrichment (BH-corrected q-value < 0.01) are counted. Both scETM and scVI-LD use latent dimensions of 100 for fair comparison.

Pathway Name	scETM	scVI-LD
REACTOME_AMYLOID_FIBER_FORMATION	1	2
KEGG_ALZHEIMERS_DISEASE	1	2
REACTOME_DEREGULATED_CDK5_TRIGGERS_MULTIPLE_ NEURODEGENERATIVE_PATHWAYS_IN_ALZHEIMER_S_DISEASE_MODELS	1	0
Total number of pathways	3	2

Table 13: Pathway enrichment statistics for AD dataset. Pathways whose names include the keywords "amyloid" or "alzheimer" are shown. For each pathway, the number of topics with significant enrichment (BH-corrected q-value < 0.01) are counted. Both scETM and scVI-LD use latent dimensions of 100 for fair comparison.

Pathway Name	scETM	scVI-LD
SUBSTANCE/DRUG INDUCED DEPRESSIVE DISORDER	2	2
Total number of pathways	1	1

Table 14: Pathway enrichment statistics for MDD dataset. Pathways whose names include the keyword "depressive" are shown. For each pathway, the number of topics with significant enrichment (BH-corrected q-value < 0.01) are counted. Both scETM and scVI-LD use latent dimensions of 100 for fair comparison.

	HP	MDD	AD
scETM	0.937	0.734	0.976
p-scETM	0.926	0.753	0.996

Table 15: Adjusted Rand Index (ARI) comparison of scETMs and p-scETMs in three human single cell transcriptomics datasets. Refer to **Incorporation of pathway knowledge into the gene embeddings in p-scETM** section in the main text for experimental details.

Pathway	Relevance	Reference
Methionine Metabolism	MDD treatment	[12]
Inflammatory mediator regulation of TRP channels	MDD treatment	[13]
Desipramine Action Pathway	MDD treatment	[14, 15]
2-arachidonoylglycerol_biosynthesis	MDD pathogenesis	[16]
transcription factor creb and its extracellular signals	MDD treatment	[17, 18]
role of erk5 in neuronal survival	MDD pathogenesis and treatment	[19]
Beta2_adrenergic_receptor_signaling	same pathway enrichment found in a MDD study	[20]
Tamoxifen Action Pathway	correlation with MDD onset	[21]
Isovaleric acidemia	correlation with MDD	[22]
Nicotine Action Pathway	correlation with MDD onset	[23]
Bumetanide Action Pathway	MDD treatment	[24]
3-Methylthiofentanyl Action Pathway	MDD treatment	[25]

Table 16: MDD-relevant pathways from the pathway-topic embedding inferred by p-scETM trained on the MDD dataset.

Pathway	Relevance	Reference
Wnt Signaling Pathway and Pluripotency	AD treatment	[26]
Alzheimer_disease_amyloid_secretase	AD pathogenesis	[27]
TNF receptor superfamily (TNFS) members mediating non-canonical NF-kB	AD treatment	[28]
Glutathione synthesis and recycling	AD pathogenesis	[29]
Endothelin_signaling	AD treatment	[30]
GABA-B_receptor_II	AD treatment	[31, 32]
GABA_Transaminase_Deficiency_Metabolite	AD treatment	[31, 32]
Leukotriene_modifiers_pathway_Pharmacodynamics	AD treatment	[33]

Table 17: AD-relevant pathways from the pathway-topic embedding inferred by p-scETM trained on the AD dataset.

	AD	MDD
DE cell-type and topics pairs	58	110
Cell types identified by DE topics	8	8
DE topics w.r.t. disease	8	15
Topics associated with cell types and disease	8	15

Table 18: Differential expression (DE) analysis summary of topics in AD and MDD data (up-regulation only).

Metric	Method	MP	HP	MDD	AD	TM
ARI	scVI-LD (10)	**0.8753**	**0.6563**	**0.6401**	**0.9889**	0.6076
	scVI-LD (100)	0.4454	0.4431	0.6145	0.9848	**0.6705**
	scVI (10)	**0.9325**	**0.7590**	0.5411	**0.9915**	**0.6699**
	scVI (100)	0.8215	0.7051	**0.5813**	0.9921	0.6284
kBET	scVI-LD (10)	0.1483	**0.0335**	**0.2759**	**0.3967**	**0.0687**
	scVI-LD (100)	**0.4601**	0.0045	0.2745	0.3570	0.0331
	scVI (10)	0.5159	**0.1403**	0.3132	0.4338	**0.0557**
	scVI (100)	**0.6856**	0.0876	**0.3402**	**0.4371**	0.0301

Table 19: Performance of scVI(-LD) with 10 and 100 latent dimensions on the five benchmark scRNA-seq datasets. The results showed that scVI and scVI-LD with 10 dimensions performed better than the same models with 100 dimensions, justifying our choice of 10 latent dimensions when comparing these models with other models. Boldface indicates which dimension choice (10 or 100) is better for the same model and dataset.

References

1. Stuart, T., Butler, A., Hoffman, P., Hafemeister, C., Papalexi, E., Mauck III, W., Hao, Y., Stoeckius, M., Smibert, P. & Satija, R. Comprehensive integration of single-cell data. *Cell*. **177**, 1888-1902 (2019)

2. Baron, M., Veres, A., Wolock, S., Faust, A., Gaujoux, R., Vetere, A., Ryu, J., Wagner, B., Shen-Orr, S., Klein, A. & Others A single-cell transcriptomic map of the human and mouse pancreas reveals inter-and intra-cell population structure. *Cell Systems*. **3**, 346-360 (2016)

3. Nagy, C., Maitra, M., Tanti, A., Suderman, M., Théroux, J., Davoli, M., Perlman, K., Yerko, V., Wang, Y., Tripathy, S. & Others Single-nucleus transcriptomics of the prefrontal cortex in major depressive disorder implicates oligodendrocyte precursor cells and excitatory neurons. *Nature Neuroscience*. pp. 1-11 (2020)

4. Mathys, H., Davila-Velderrain, J., Peng, Z., Gao, F., Mohammadi, S., Young, J., Menon, M., He, L., Abdurrob, F., Jiang, X. & Others Single-cell transcriptomic analysis of Alzheimer's disease. *Nature*. **570**, 332-337 (2019)

5. Consortium, T. & Others Single-cell transcriptomics of 20 mouse organs creates a Tabula Muris.. *Nature*. **562**, 367 (2018)

6. Sunkin, S., Ng, L., Lau, C., Dolbeare, T., Gilbert, T., Thompson, C., Hawrylycz, M. & Dang, C. Allen Brain Atlas: an integrated spatio-temporal portal for exploring the central nervous system. *Nucleic Acids Research*. **41**, D996-D1008 (2012)

7. Macosko, E., Basu, A., Satija, R., Nemesh, J., Shekhar, K., Goldman, M., Tirosh, I., Bialas, A., Kamitaki, N., Martersteck, E., Trombetta, J., Weitz, D., Sanes, J., Shalek, A., Regev, A. & McCarroll, S. Highly Parallel Genome-wide Expression Profiling of Individual Cells Using Nanoliter Droplets. *Cell*. **161**, 1202-1214 (2015)

8. Shekhar, K., Lapan, S., Whitney, I., Tran, N., Macosko, E., Kowalczyk, M., Adiconis, X., Levin, J., Nemesh, J., Goldman, M., McCarroll, S., Cepko, C., Regev, A. & Sanes, J. Comprehensive Classification of Retinal Bipolar Neurons by Single-Cell Transcriptomics. *Cell*. **166**, 1308-1323.e30 (2016)

9. Butler, A., Hoffman, P., Smibert, P., Papalexi, E. & Satija, R. Integrating single-cell transcriptomic data across different conditions, technologies, and species. *Nature Biotechnology*. **36**, 411-420 (2018)

10. Seurat3.0 finding integration vectors: long vectors not supported yet number 1029. `https://github.com/satijalab/seurat/issues/1029`. accessed 5 jan 2021.

11. Traag, V., Waltman, L. & Eck, N. From Louvain to Leiden: guaranteeing well-connected communities. *Scientific Reports*. **9** pp. 5233 (2019,3)

12. De Berardis, D., Orsolini, L., Serroni, N., Girinelli, G., Iasevoli, F., Tomasetti, C., Bartolomeis, A., Mazza, M., Valchera, A., Fornaro, M. & Others. A comprehensive review on the efficacy of S-adenosyl-L-methionine in major depressive disorder. *CNS & Neurological Disorders-Drug Targets (Formerly Current Drug Targets-CNS & Neurological Disorders)*. **15**, 35-44 (2016)

13. Chahl, L. TRP channels and psychiatric disorders. *Transient Receptor Potential Channels*. pp. 987-1009 (2011)

14. Nelson, J., Mazure, C. & Jatlow, P. Desipramine treatment of major depression in patients over 75 years of age. *Journal Of Clinical Psychopharmacology*. **15**, 99-105 (1995)

15. Nelson, J. Use of desipramine in depressed inpatients.. *The Journal Of Clinical Psychiatry*. (1984)

16. Hill, M., Miller, G., Ho, W., Gorzalka, B. & Hillard, C. Serum endocannabinoid content is altered in females with depressive disorders: a preliminary report. *Pharmacopsychiatry*. **41**, 48 (2008)

17. Blendy, J. The role of CREB in depression and antidepressant treatment. *Biological Psychiatry*. **59**, 1144-1150 (2006)

18. Koch, J., Kell, S., Hinze-Selch, D. & Aldenhoff, J. Changes in CREB-phosphorylation during recovery from major depression. *Journal Of Psychiatric Research*. **36**, 369-375 (2002)

19. Wang, J. & Mao, L. The ERK pathway: molecular mechanisms and treatment of depression. *Molecular Neurobiology*. **56**, 6197-6205 (2019)

20. Qiu, A., Shen, M., Buss, C., Chong, Y., Kwek, K., Saw, S., Gluckman, P., Wadhwa, P., Entringer, S., Styner, M. & Others Effects of antenatal maternal depressive symptoms and socio-economic status on neonatal brain development are modulated by genetic risk. *Cerebral Cortex*. **27**, 3080-3092 (2017)

21. Day, R., Ganz, P. & Costantino, J. Tamoxifen and depression: more evidence from the National Surgical Adjuvant Breast and Bowel Project's breast cancer prevention (P-1) randomized study. *Journal Of The National Cancer Institute*. **93**, 1615-1623 (2001)

22. Szczesniak, O., Hestad, K., Hanssen, J. & Rudi, K. Isovaleric acid in stool correlates with human depression. *Nutritional Neuroscience*. **19**, 279-283 (2016)

23. Breslau, N., Kilbey, M. & Andreski, P. Nicotine dependence, major depression, and anxiety in young adults. *Archives Of General Psychiatry*. **48**, 1069-1074 (1991)

24. Goubert, E., Altvater, M., Rovira, M., Khalilov, I., Mazzarino, M., Sebastiani, A., Schaefer, M., Rivera, C. & Pellegrino, C. Bumetanide prevents brain trauma-induced depressive-like behavior. *Frontiers In Molecular Neuroscience*. **12** pp. 12 (2019)

25. Ehrich, E., Turncliff, R., Du, Y., Leigh-Pemberton, R., Fernandez, E., Jones, R. & Fava, M. Evaluation of opioid modulation in major depressive disorder. *Neuropsychopharmacology*. **40**, 1448-1455 (2015)

26. De Ferrari, G. & Inestrosa, N. Wnt signaling function in Alzheimer's disease. *Brain Research Reviews*. **33**, 1-12 (2000)

27. Holsinger, R., McLean, C., Beyreuther, K., Masters, C. & Evin, G. Increased expression of the amyloid precursor β-secretase in Alzheimer's disease. *Annals Of Neurology: Official Journal Of The American Neurological Association And The Child Neurology Society*. **51**, 783-786 (2002)

28. Decourt, B., K Lahiri, D. & N Sabbagh, M. Targeting tumor necrosis factor alpha for Alzheimer's disease. *Current Alzheimer Research*. **14**, 412-425 (2017)

29. Saharan, S. & Mandal, P. The emerging role of glutathione in Alzheimer's disease. *Journal Of Alzheimer's Disease*. **40**, 519-529 (2014)

30. Palmer, J., Barker, R., Kehoe, P. & Love, S. Endothelin-1 is elevated in Alzheimer's disease and upregulated by amyloid-β. *Journal Of Alzheimer's Disease*. **29**, 853-861 (2012)

31. Li, Y., Sun, H., Chen, Z., Xu, H., Bu, G. & Zheng, H. Implications of GABAergic neurotransmission in Alzheimer's disease. *Frontiers In Aging Neuroscience*. **8** pp. 31 (2016)

32. Govindpani, K., Calvo-Flores Guzmán, B., Vinnakota, C., Waldvogel, H., Faull, R. & Kwakowsky, A. Towards a better understanding of GABAergic remodeling in Alzheimer's disease. *International Journal Of Molecular Sciences*. **18**, 1813 (2017)

33. Michael, J., Marschallinger, J. & Aigner, L. The leukotriene signaling pathway: a druggable target in Alzheimer's disease. *Drug Discovery Today*. **24**, 505-516 (2019)
