# Supplementary material for: Learning interpretable cellular and gene signature embeddings from single-cell transcriptomic data
Source: Nat Commun. 2021 Sep 6;12:5261. doi: 10.1038/s41467-021-25534-2 (PMC8421403; doi:10.1038/s41467-021-25534-2)
Supplement: Supplementary file 3 — Description of Additional Supplementary Files [file 41467_2021_25534_MOESM3_ESM.pdf]

**Description of Additional Supplementary Files**

File Name: Supplementary  Data 1
Description: Pathway enrichment statistics breakdown of scETM for four datasets including HP, AD, MDD (all genes) and MDD (coding genes only), and scVI-LD for three datasets including HP, AD, MDD (all genes).

File Name: Supplementary Data 2
Description: Top 5 pathways of each topic (row) based on alpha intensities for four datasets including HP, AD MDD (all genes), MDD (coding genes only).
