# Supplementary material for: Learning interpretable cellular and gene signature embeddings from single-cell transcriptomic data
Source: Nat Commun. 2021 Sep 6;12:5261. doi: 10.1038/s41467-021-25534-2 (PMC8421403; doi:10.1038/s41467-021-25534-2)
Supplement: Supplementary file 6 — Reporting Summary [file 41467_2021_25534_MOESM6_ESM.pdf]

nature portfolio

Corresponding author(s): Yue Li, Jian Tang

Last updated by author(s): Aug 11, 2021

Reporting Summary

Nature Portfolio wishes to improve the reproducibility of the work that we publish. This form provides structure for consistency and transparency in reporting. For further information on Nature Portfolio policies, see our Editorial Policies and the Editorial Policy Checklist.

Statistics

For all statistical analyses, confirm that the following items are present in the figure legend, table legend, main text, or Methods section.

n/a	Confirmed	
☐	☒	The exact sample size (*n*) for each experimental group/condition, given as a discrete number and unit of measurement
☐	☒	A statement on whether measurements were taken from distinct samples or whether the same sample was measured repeatedly
☐	☒	The statistical test(s) used AND whether they are one- or two-sided<br>*Only common tests should be described solely by name; describe more complex techniques in the Methods section.*
☐	☒	A description of all covariates tested
☐	☒	A description of any assumptions or corrections, such as tests of normality and adjustment for multiple comparisons
☐	☒	A full description of the statistical parameters including central tendency (e.g. means) or other basic estimates (e.g. regression coefficient) AND variation (e.g. standard deviation) or associated estimates of uncertainty (e.g. confidence intervals)
☐	☒	For null hypothesis testing, the test statistic (e.g. *F*, *t*, *r*) with confidence intervals, effect sizes, degrees of freedom and *P* value noted<br>*Give P values as exact values whenever suitable.*
☐	☒	For Bayesian analysis, information on the choice of priors and Markov chain Monte Carlo settings
☐	☒	For hierarchical and complex designs, identification of the appropriate level for tests and full reporting of outcomes
☒	☐	Estimates of effect sizes (e.g. Cohen's *d*, Pearson's *r*), indicating how they were calculated

Our web collection on statistics for biologists contains articles on many of the points above.

Software and code

Policy information about availability of computer code

Data collection	No software used to collect the data. All single-cell transcriptome data were downloaded from the corresponding websites as described in Supplementary section S1.1 Data processing.
Data analysis	R (v4.0.2) packages: Seurat (v3.2.2), SeuratDisk (v0.0.0.9103), SeuratWrappers (v0.3.0), dplyr (v1.0.2), cowplot (v1.1.0), argparse (v2.0.1), aricode (v1.0.0), reticulate (v1.18), rliger (v0.5.0), ComplexHeatmap (v2.4.3), circlize (v0.4.12), cluster (v2.1.1), svglite (v2.0.0);<br>Python (v3.7.4) packages: scETM (v0.4.9, https://github.com/hui2000ji/scETM), scvi (v0.6.5), scvae (v2.1.4), scanorama (v1.6), harmonypy (v0.0.5), psutil (v5.6.7), PyTorch (v1.5), numpy (v1.16.2), scikit-learn (v0.20.3), h5py (v2.9.0), pandas (v0.25), tqdm (v4.31.1), anndata (v0.7), scanpy (v1.4.6), scipy (v1.0), louvain (v0.6.1), leidenalg (v0.7.0), seaborn (v0.10.0), matplotlib (v3.1.2);<br>GSEA (v4.1.0).

For manuscripts utilizing custom algorithms or software that are central to the research but not yet described in published literature, software must be made available to editors and reviewers. We strongly encourage code deposition in a community repository (e.g. GitHub). See the Nature Portfolio guidelines for submitting code & software for further information.

Data

Policy information about availability of data

All manuscripts must include a data availability statement. This statement should provide the following information, where applicable:

- Accession codes, unique identifiers, or web links for publicly available datasets
- A description of any restrictions on data availability
- For clinical datasets or third party data, please ensure that the statement adheres to our policy

The datasets analyzed during the current study are from publicly available repositories or data portals. The acquisition and quality control steps for all datasets are

included in the supplementary information. The Human pancreatic islet dataset used in this study are available in the GEO or EMBL-EBI database under the accession codes GSE81076 (https://www.ncbi.nlm.nih.gov/geo/query/acc.cgi?acc=GSE81076), GSE85241 (https://www.ncbi.nlm.nih.gov/geo/query/acc.cgi?acc=GSE85241), GSE86469 (https://www.ncbi.nlm.nih.gov/geo/query/acc.cgi?acc=GSE86469), E-MTAB-5061 (https://www.ebi.ac.uk/arrayexpress/experiments/E-MTAB-5061/), and GSE84133 (https://www.ncbi.nlm.nih.gov/geo/query/acc.cgi?acc=GSE84133).
Mouse pancreatic islet dataset is available in the GEO database under the accession code GSE84133 (https://www.ncbi.nlm.nih.gov/geo/query/acc.cgi?acc=GSE84133).
Major Depressive Disorder dataset is available in the GEO database under the accession code GSE144136 (https://www.ncbi.nlm.nih.gov/geo/query/acc.cgi?acc=GSE144136).
Mouse Retina dataset is available in the GEO database under the accession codes GSE63473 (https://www.ncbi.nlm.nih.gov/geo/query/acc.cgi?acc=GSE63473) and GSE81904 (https://www.ncbi.nlm.nih.gov/geo/query/acc.cgi?acc=GSE81904).
The Alzheimer's Disease (AD) is available in Synapse under the access code syn18485175 (https://www.synapse.org/Synapse:syn18485175).
The Tabula Muris dataset is available in the FigShare database under the accession code 27733 (https://figshare.com/projects/Tabula_Muris_Transcriptomic_characterization_of_20_organs_and_tissues_from_Mus_musculus_at_single_cell_resolution/27733).
The Allen Brain Atlas datasets namely human primary motor cortex and the primary motor cortex datasets and are available from the Allen Brain Portal (https://portal.brain-map.org/atlases-and-data/rnaseq/human-m1-10x, https://portal.brain-map.org/atlases-and-data/rnaseq/mouse-whole-cortex-and-hippocampus-10x, respectively).

Field-specific reporting

Please select the one below that is the best fit for your research. If you are not sure, read the appropriate sections before making your selection.

☒ Life sciences ☐ Behavioural & social sciences ☐ Ecological, evolutionary & environmental sciences

For a reference copy of the document with all sections, see nature.com/documents/nr-reporting-summary-flat.pdf

Life sciences study design

All studies must disclose on these points even when the disclosure is negative.

Sample size	We analyzed eight datasets in this study. To assess scETM's clustering performance with other state-of-the-art methods, we used six datasets of varying sizes, that is, (1) Mouse Pancreatic Islet (MP, n=1,886); (2) Mouse Retina (MR, n=72,307); (3) Tabula Muris (TM-FACS, n=44,879; TM-droplet, n=55,656), (4) Human Pancreatic Islet (HP, n=14,890); (5) Alzheimer's Disease dataset (AD, n=70,634), and (6) Major Depressive Disorder dataset (MDD, n=78,886). We next investigated the batch correction performance using HP and MR, which consist of 5 and 2 subsets from independent empirical studies, respectively. For the scalability benchmark, we merged AD and MDD and randomly sampled 28,000, 14,000, 70,000 and 148,247 cells. We then demonstrated scETM's cross-species and cross-tissue transfer learning performance using MP, TM-FACS, HP, plus two Allen Brain Altas datasets, namely (7) Human primary motor cortex (HumM1C, n=76,533) and (8) Mouse primary motor area (MusMOp, n=1,093,785). Lastly, we conducted exploratory analysis on the scETM and pathway-scETM topics using HP, AD, and MDD. In total, we analyzed over 1.5M cells collected from 14 studies from two species. Thus, we believe the sample size is sufficient for testing our algorithm.
Data exclusions	For quality control, we followed the same steps as in the respective original empirical studies. Below we list the additional steps taken in the current study. For MusMOp and HumM1C, we excluded the cells and nuclei with subclass label "outlier". Only cells with the region label "MOp" (n=1,093,785) were kept in the MusMOp dataset. For MR, we kept the genes shared by the two subsets, and filtered out the 669 samples labeled as "Doublets/Contaminants" in the Shekhar et al. batch. The merged dataset contains 71,638 cells and 12333 genes. In the scalability analysis, we used the 3000 most variable genes in the MDD-AD merged dataset while varying the sample sizes.
Replication	All findings were produced by computer program and fully reproducible. Although repeated runs produce slightly different embeddings, the results are reproducible in terms of discerning different cell types and disease-specific patterns.
Randomization	The allocation was random. Some datasets have specific ascertainment biases, e.g., both Major depressive disorder dataset and Alzheimer's Disease dataset contain half healthy and half disease brain samples.
Blinding	The investigators were blinded to the group allocation during the data collection and analysis.

Reporting for specific materials, systems and methods

We require information from authors about some types of materials, experimental systems and methods used in many studies. Here, indicate whether each material, system or method listed is relevant to your study. If you are not sure if a list item applies to your research, read the appropriate section before selecting a response.

Materials & experimental systems

n/a	Involved in the study
☒	☐ Antibodies
☒	☐ Eukaryotic cell lines
☒	☐ Palaeontology and archaeology
☒	☐ Animals and other organisms
☒	☐ Human research participants
☒	☐ Clinical data
☒	☐ Dual use research of concern

Methods

n/a	Involved in the study
☒	☐ ChIP-seq
☒	☐ Flow cytometry
☒	☐ MRI-based neuroimaging
